# Hybrid artificial electric field employing cuckoo search algorithm with refraction learning for engineering optimization problems

**DOI:** 10.1038/s41598-023-31081-1

**Published:** 2023-03-12

**Authors:** Oluwatayomi Rereloluwa Adegboye, Ezgi Deniz Ülker

**Affiliations:** 1 Computer Engineering Department, European University of Lefke, Mersin-10, Turkey; 2 Software Engineering Department, European University of Lefke, Mersin-10, Turkey

**Keywords:** Computational science, Computer science

## Abstract

Due to its low dependency on the control parameters and straightforward operations, the Artificial Electric Field Algorithm (AEFA) has drawn much interest; yet, it still has slow convergence and low solution precision. In this research, a hybrid Artificial Electric Field Employing Cuckoo Search Algorithm with Refraction Learning (AEFA-CSR) is suggested as a better version of the AEFA to address the aforementioned issues. The Cuckoo Search (CS) method is added to the algorithm to boost convergence and diversity which may improve global exploration. Refraction learning (RL) is utilized to enhance the lead agent which can help it to advance toward the global optimum and improve local exploitation potential with each iteration. Tests are run on 20 benchmark functions to gauge the proposed algorithm's efficiency. In order to compare it with the other well-studied metaheuristic algorithms, Wilcoxon rank-sum tests and Friedman tests with 5% significance level are used. In order to evaluate the algorithm’s efficiency and usability, some significant tests are carried out. As a result, the overall effectiveness of the algorithm with different dimensions and populations varied between 61.53 and 90.0% by overcoming all the compared algorithms. Regarding the promising results, a set of engineering problems are investigated for a further validation of our methodology. The results proved that AEFA-CSR is a solid optimizer with its satisfactory performance.

## Introduction

In real world applications, optimization problems are frequently non-differentiable, non-convex and discontinuous. Before the introduction of the most extensively used metaheuristic optimization technique, gradient descent approach was one of the optimization techniques employed as well as the Gauss–Newton technique^1,2^. The gradient-based optimization method is vulnerable to getting over the local optimums and reduces the precision of optimization. On the other hand, metaheuristic optimization algorithms are able to find ideal or nearly ideal solutions in a manageable time. These algorithms have been studied by many researchers in order to deal with difficult optimization problems, some of them are Genetic Algorithm (GA)^3^, Particle Swarm Optimization (PSO)^4^, Hybrid Artificial Humming Bird-Simulated Annealing (HAHA-SA)^5^, Differential Evolution (DE)^6^, Hybrid Flow Direction Optimizer-dynamic oppositional based algorithm (HFDO-DOBL)^7^, Firefly Algorithm (FA)^8^, Artificial Electric Field Algorithm (AEFA)^9,10^, Artificial Bee Colony Optimization (ABC)^11^, Hybrid Heat Transfer Search and Passing Vehicle Search Optimizer (MOHHTS–PVS)^12^, Cuckoo Search (CS)^13^, Chaotic Marine Predators Algorithm (CMPA)^14^ and Nelder Mead-infused INFO algorithm (HINFO-NM)^15^. AEFA has become the focus of research among these algorithms in recent years with its few parameters and its simple principle.

AEFA is a stochastic optimization algorithm based on swarm intelligence. Due to the interaction between the charged particles via electrostatic force; attraction or repulsion. The particles travel along the electrostatic field with the most charged particle leading. When it was first introduced, academics were intrigued by the efficiency of AEFA. It has been used extensively in numerous fields, such as machine learning^16,17^, assembly lines^18^, engineering problems^19,20^, feature selection^21^ and economic load dispatch problem^22^. The leading charged particle controls each iteration of the search process of AEFA.

In multimodal problems, the leading charged particle may occasionally enter a sub-optimal location^23^. The population is nevertheless subject to local optimal, when the leading charged particle becomes stranded in a sub-optimal location. Less diversity in population becomes inevitable because of the significant convergence of other particles toward the leading charged particle. Consequently, the standard AEFA has the similar problems as the majority of metaheuristic algorithms such as; lack of population variety and tendency to be trapped in local optimum points.

The aforementioned problems are the main motivation of this study. Therefore, a hybridized version of Artificial Electric Field Algorithm (AEFA) with Cuckoo Search (CS) using Refraction Learning (RL) is proposed and called AEFA-CSR. In this hybrid version, two search methods with different properties are introduced to provide new solutions in the population. One of them makes use of the idea of light refraction to learn opposite solution. This method is proposed in order to enhance the lead particle search functionality and broaden its range to avoid sub-optimality. Additionally, the CS method is used to increase population variety. By weakening the leadership of the leading particle and allowing particles to identify viable solutions will enable particles to be moved from other sub-optimal solutions. Using the aforementioned strategies in combination, the performance of AEFA-CSR becomes quite noticeable.

There are many AEFA variations in the literature. However, to the best of our knowledge, AEFA-CSR is the first variant of AEFA that employs CS and RL. In order to overcome the limitations of AEFA, CS is chosen in particular for enhancing the population diversity and RL was specifically applied for increasing the ability of escaping from multiple local optimums. These features assess the AEFA-CSR to perform contributions to the field with an enhanced solution quality and for real-world engineering problems. The steps are specifically investigated to obtain enhanced and strengthened features in order to overcome the limitations of AEFA.

The rest of the paper is structured as follows. In Section “Related work”, related metaheuristic optimization techniques, in particular AEFA variations and their contributions are given. In Section “Methodology”, the methodology used to form AEFA-CSR is discussed. The algorithms; AEFA, CS, RL are elaborated individually by mentioning the main motivation of this study. In Section “Experimental results”, the experimental results obtained by the proposed algorithm AEFA-CSR with the counterpart algorithms such as well-studied and commonly used ones, recently developed ones and hybrid ones for the benchmark functions are presented. Additionally, some particular tests to observe the efficiency of AEFA-CSR are employed and the results are discussed. Moreover, to measure the suitability of the algorithm for real world engineering design problems, some problems are studied and analyzed. Finally, in Section “Conclusion and future work”, the concluding statements and the future work are given.

## Related work

There is a widespread use of metaheuristic optimization techniques. These techniques are used to address optimization challenges and can be categorized into three groups: physics-inspired algorithms, evolution-inspired algorithms and swarm-inspired algorithms. Physics-inspired algorithms imitate the physical laws that govern how individuals engage with each other and their search space. These laws include the laws of inertia, light refraction, gravitation and many others^24^. Few of the popular algorithms in this category are Gravitational Search Algorithm (GSA)^25^, Colliding Bodies Optimization (CBO)^26^ and Henry Gas Solubility Optimization (HGSO)^27^. Evolution-inspired algorithms simulate the natural process of evolution by trying different combinations of individuals to find the best solution. The best individuals are combined to form a new generation, which is the key advantage of this technique. Few of these algorithms are Genetic Algorithm (GA)^3^, Differential Evolution (DE)^6^ and Evolutionary Programming (EP)^28^. Swarm-inspired algorithms aim to develop intelligent swarm behaviors like animal grazing and bird flocking. In the search space, promising areas will be discovered by a population that collaborate and interact. Few of the recent swarm-inspired algorithms are Whale Optimization Algorithm (WOA)^29^, Seagull Optimization Algorithm (SOA)^30^, Particle Swarm Optimization (PSO)^4^, and Harris Hawks Optimization (HHO)^31^.

Each of the numerous metaheuristic algorithms has its own drawbacks. The local and global search balance in Grey Wolf Optimizer (GWO) is weak^24^. Rodríguez et al. investigated the possibility of altering the initial control parameters to improve the exploration process in GWO^32^. GWO does not have an effective diverse population, so Lou et al. switched from the typical real-valued encoding method to a complex-valued one which makes the population more diverse^33^. WOA easily enters the local optimum and suffers from premature convergence. Using chaotic maps, Oliva et al. modified WOA to prevent the population from entering local optima^34^. Shi et al. proposed a new chaos-based operator and a new neighbor selection strategy to speed up the convergence of Artificial Bee Colony (ABC) both of which improved the standard ABC^35^.

AEFA is a type of physics-inspired algorithm that mimics the group of particles interact and move along the search space. It is simple to implement and has fewer parameters. As a result, a variety of optimization problems have been successfully solved using this algorithm. Controller design^36^, multi-objective optimization problems^37^, soil shear strength prediction^38^, pattern search ^39^, vehicle routing ^40^ and tumor detection ^17^ are some of the examples of the research problems solved by AEFA. To improve performance and address the shortcomings of the AEFA, numerous academics have developed variants of the original AEFA in recent years. Malisetti and Pamula used Moth Levy methodology to create Moth Levy Artificial Electrical Field Algorithm (ML-AEFA) to solve the problem of entering the sub-optimality^41^. An algorithm with new strategy for velocity update and population initialization known as improved Artificial Electrical Field Algorithm (IAEFA) has been introduced in order to enhance the robustness of AEFA in handling complex problems^42^. Furthermore, due to the algorithm’s focus primarily on local search, it is unable to effectively perform efficient global exploration across the entire solution space. Cheng et al. used a log-sigmoid function in order to strike a balance between exploration and exploitation^16^. AEFA with inertia and repulsion known as improved Artificial Electrical Field Algorithm (IAEFA) is introduced by Bi et al. to avoid premature convergence and improve population diversity^43^. Modified Artificial Electrical Field Algorithm (mAEFA) is proposed by Houssein et al.^23^. Levy flight, local escaping operator and opposition learning are introduced to avert stagnation in regions of local optimal^23^. Extensive experiment shows improvement in convergence rate and search ability of AEFA. To attain better exploitation and exploration balance, Anita, Yadav and Kumar introduced AEFA for solving constrained optimization problems (AEFA-C) by constraining particle interaction to the search space's border using new velocity and location updates^19^. The improved version of the AEFA that was proposed by Demirören et al. as Opposition based Artificial Electrical Field Algorithm (ObAEFA) makes use of the opposition-based learning strategy to improve the AEFA exploration capabilities^36^. The improved performance of ObAEFA was vetted through several experiments. Petwal and Rani’s experimental findings show that the proposed algorithm is highly competitive and achieves the desired level of population diversity^37^. AEFA based on opposition learning is proposed to enhance its global exploration and local development capabilities. The opposition learning strategy is used to increase population diversity and exploitation, while the chaos strategy is used to improve the quality of the initial population, experiments demonstrate the algorithm's superior performance^44^. Furthermore, Levy flight mechanism that provides multiple distinct evolutionary strategies and enhances the local search capability was introduced to AEFA by Sinthia and Malathi^17^. The elitism selection theory ensures that the fittest survive and mutation operators increase population diversity. The performance of the multi strategy Artificial Electrical Field Algorithm (M-AEFA) is enhanced by the dynamic combination of these adaptive strategies.

Hybridizing AEFA with other types of algorithms like swarm-inspired or physics-inspired algorithms to enhance the performance of AEFA is another area of research interest. The poor exploitation as a result of the stochastic nature of AEFA is be improved by hybridizing AEFA with Nelder-Mead (NM) simplex. AEFA performs the global search while NM performs the local search. Test on popular functions show improved performance^20^. One of the well-known optimization algorithms DE is applied to create an effective hybrid by combining the capabilities of AEFA and DE (AEFA-DE). On IEEE Congress on Evolutionary Computation-2019 (CEC-2019) test functions, the performance of the suggested hybrid method is validated. The experimental findings imply that AEFA-DE performs better than the compared algorithms^45^.

AEFA is able to conduct a more in-depth search across the solution space using the local search mechanism^18^. It was discovered that the location of the charged particle and the mutual attraction of the nearby particles influence how the artificial electric field algorithm updates its position. Despite of the strong local search ability of AEFA, it has limited global search capacity. It is because of the charged particles have a strong ability to interact with information. The Sine–Cosine Algorithm (SCA) can better balance local and global search than AEFA. As a result, SCA’s update mechanism is included into the AEFA (SC-AEFA) by changing the iterative process of the algorithm^40^.

The major goal of the derivatives of AEFA is to increase search accuracy and convergence speed, in accordance with the numerous improvement methodologies indicated above. As a result, this study presents the AEFA-CSR, an improved Artificial Electric Field Algorithm based on Cuckoo Search (CS) with Refraction Learning (RL).

## Methodology

### Artificial electrical field algorithm

The Coulomb's law states that "electrostatic forces of repulsion or attraction among two different charge particles are in direct proportion with the product of charges and in inverse proportion with the square of the distances between their positions". This idea serves as the basis for the limitation of AEFA^9^. In this case, the charged particles are referred to as agents and the charges of particles are used to evaluate the agents' potentials. All charged particles may experience an electrostatic force of either repelling or attracting as a result of the movement of objects in the search space. The charges use electrostatic forces to communicate directly and their positions provide the best solution. As a result, charges are referred to as a function of the population's fitness and the potential solution. The electrostatic force of attraction states that the charged particles with the least charge are drawn to the charged particles with the most charge. In addition, the solution with the highest charge is thought to be the best^46^. The pseudocode of AEFA can be seen in Algorithm 1.

Assuming ${Y}_{j}=\left({Y}_{j}^{1},{Y}_{j}^{2},\dots ,{{Y}_{j}^{{ D}_{N}}}\right)\forall j=1,2 ,\dots ,N$ where the *j*th particle has dimension ${D}_{N}$. By employing the location and best personal fitness value of particular particle, that particle is able offer the best global fitness value in AEFA. To acquire the optimum position fitness value of any particle $j$ throughout interval formulas are expressed below^43^,

1 $$\begin{array}{c}\\ {B}_{j}^{{D}_{N}}(t+1)=\left\{\begin{array}{ll}{B}_{j}^{{D}_{N}}(t)& \text{if fitness }\left({B}_{j}^{{D}_{N}}(t)\right)<{\mathrm{fit}}\left({Y}_{j}(t+1)\right)\\ {Y}_{j}(t+1)& \text{if fitness }\left({Y}_{j}(t+1)\le {\mathrm{fit}}\left({B}_{j}(t)\right)\right.\end{array}\right.\end{array}$$

where a particle’s personal best fitness and current position are represented as ${B}_{j}$ and ${Y}_{j}$ respectively. Furthermore, the force on the charge $l$ exacted by $j$ throughout interval $I$ is given in the following Eq. (2)^9^,

2 $${\text{Force }}_{jl}^{{D}_{N}}(t)=K(t)\frac{{q}_{j}(t)\times {q}_{l}(t)\left({B}_{l}^{{D}_{N}}(t)-{Y}_{j}^{{D}_{N}}(t)\right)}{{DIST}_{jl}(t)+\varepsilon }$$

where ${q}_{l}$ and ${q}_{j}$ are charge of any particle $l$ and $j$ is expressed as,

3 $${q}_{l}\left(t\right)=\mathrm{exp}\left(\frac{\text{ Fitness }\left({B}_{l}\right)(t)-\mathrm{Worst}(t)}{\mathrm{Best}(t)-\mathrm{Worst}(t)}\right)$$

4 $$\left\{\begin{array}{c}\mathrm{Best}\left(t\right)=\mathrm{min}\left({\text{ Fitness }}_{l}\left(t\right)\right) l=(\mathrm{1,2},\dots ,N)\\ \text{ Worst }\left(t\right)=\mathrm{max}\left({\text{ Fitness }}_{l}\left(t\right)\right) l=(\mathrm{1,2},\dots ,N)\end{array}\right\}$$

$\mathrm{Worst} \left(t\right)$ and $\mathrm{Best}(t)$ represent the worst and best fitness among all charges. $K(t)$ and $\varepsilon$ denote the Coulomb’s constant and a positive epsilon constant, respectively. The Euclidean distances between two independent particles at interval $t$ is therefore represented as ${DIST}_{jl}(t)$ and are calculated as follows.

5 $${DIST}_{jl}(t)= \left\| {{Y}_{j}(t),{Y}_{l}(t)}_{2} \right\| $$

In addition, Eq. (6) gives the assessment of ${\text{max }}_{\text{iter}}$ and current ${\text{iteration}}$ with respect to the Coulombs rule. The parameters of the Coulombs rule are represented by ${K}_{0}$ and $\gamma$ respectively.

6 $$K(t) ={K}_{0}\times \mathrm{exp}\left(-\gamma \frac{\text{ iter }}{{\text{ max }}_{\text{iter}}}\right)$$

where ${\text{max }}_{\text{iter}}$ refers to total number of iteration preset at the beginning and *iter* is the current iteration number when computing Coulomb’s constant.

The total electric force on $jth$ particle with the dimension ${D}_{N}$ is thus stated as follows,

7 $${\text{Total Force }}_{jl}^{{D}_{N}}\left(t\right)=\sum_{l=1,l\ne j}^{N} R\times \left[{\text{ Force }}_{jl}^{{D}_{N}}(t)\right]$$

where $R$ depict random number from the range of [0–1]. Individual charge divided by total individual charge of all particles is expressed as ${Q}_{l}\left(t\right)$ as follows.

8 $${Q}_{l}\left(t\right)=\frac{{q}_{l}(t)}{\sum_{l=1}^{N} {q}_{l}(t)}$$

Equations (9) and (10) describe the equations for the respective electric field $E{F}_{j}^{{D}_{N}}(t)$ and acceleration ${\mathrm{Acc}}_{j}^{{D}_{N}}(t)$ of the *j*th particle having the dimension as ${D}_{N}$ over interval $t$,

9 $$E{F}_{j}^{{D}_{N}}(t)=\frac{{\text{ Total \, Force }}_{jl}^{{D}_{N}}(t)}{{Q}_{l}\left(t\right)}$$

10 $${\mathrm{Acc}}_{j}^{{D}_{N}}(t)=\frac{{Q}_{j}\left(t\right)\times E{F}_{j}^{{D}_{N}}(t)}{M{a}_{j}(t)}$$

where $M{a}_{j}\left(t\right)$ represent the mass of particle $j.$ Equations (11) and (12) provide the update equations for the velocity and location of the *j*th particle as follows,

11 $${V}_{j}^{{D}_{N}}(t+1)=R\times {V}_{j}^{{D}_{N}}(t)+{\mathrm{Acc}}_{j}^{{D}_{N}}(t)$$

12 $${Y}_{j}^{{D}_{N}}(t+1)={Y}_{j}^{{D}_{N}}(t)+{V}_{j}^{{D}_{N}}(t+1)$$

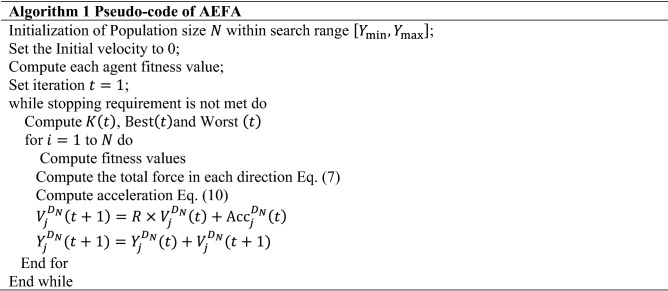


### Cuckoo search

The strong reproductive strategy of some cuckoo species encourages the idea of Cuckoo Search (CS)^13,47^ which is a type of metaheuristic algorithm inspired by the swarm intelligence. Three rules regulate CS operations and the last rule entails adding some fresh random solutions to the process^48^. An approximation of it is a fraction $Pa$ of the $n$ number of host nests to create new nests. The basic steps of the CS can be determined by following the cuckoo breeding behavior, which can be found in^49^ and was summed up in^50^. The optimization issue to be tackled is portrayed as $f(Y)$ where nests are represented as $Y=$ $\left\{{Y}_{1},{Y}_{2}, {Y}_{3},{\dots Y}_{D}\right\}$ with $D$ dimensions. Within the designated search space there are $N$ host nests $\left\{{Y}_{i},i=1,\dots ,N\right\}$. Each of the ${Y}_{i}=\left\{{Y}_{i1},\dots ,{Y}_{iD}\right\}$ nests indicates a potential solution to the optimization task at hand. Finding the new population of ${Y}_{i}(t+1)$ nests is one of the algorithm’s crucial phases. Additionally, the following equation shows the use of the Levy flight to gain the new nests at a time $t$,

13 $${Y}_{ij}(t+1)={Y}_{ij}(t)+\alpha \oplus \mathrm{Le}vy(\lambda )$$

where $\lambda$ is a Lévy flight parameter, $\oplus$ is an entry-wise multiplication operation, and $\alpha$ is the step size. As aforementioned the third rule imitate this notion, host birds will abandon nest given alien eggs are found. In this case, the following method can be used to regenerate new nest with probability $Pa$,

14 $${Y}_{i}\left(t+1\right)={Y}_{i}\left(t\right)+R\times \left({Y}_{y}\left(t\right)-{Y}_{j}\left(t\right)\right)$$

where two randomly chosen nest from host of nests are ${Y}_{y}\left(t\right)$ and ${Y}_{j}\left(t\right)$. $R$ is a random value between [0,1]. The step by step execution of CS can be seen in Algorithm 2.


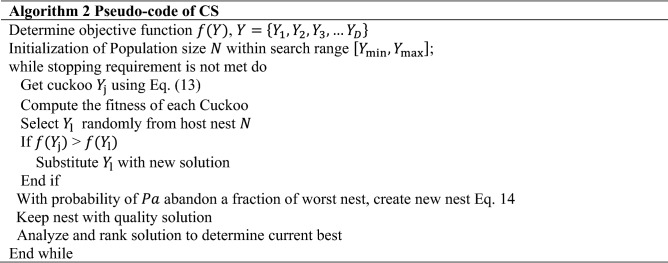


### Artificial electric field employing Cuckoo search algorithm with Refraction learning (AEFA-CSR)

AEFA-CSR algorithm has been proposed with the proper use of the previous techniques. The algorithm does not only combine the benefits of the Artificial Electric Field Algorithm (AEFA) but two of the algorithms; Cuckoo Search (CS) and Refraction Learning (RL) as well as incorporating a sub-optimal avoidance technique. This offers quite noticeable global search capabilities as well as the ability to avoid being trapped into local optimum points. In the Algorithm 3, the detailed steps of AEFA-CSR are given.
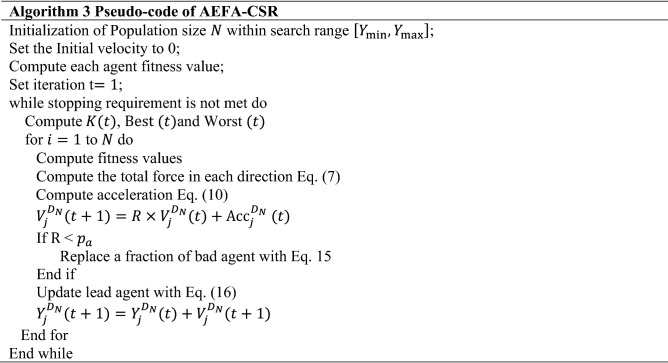


#### Motivation

An algorithm may become inefficient, if the exploration ability performs excessive. In the same way excessive exploitation may trap the algorithm in sub-optimal prematurely and may provide unacceptable results. Consequently, the balance between exploration and exploitation is crucial for an algorithm’s efficiency^51^. It was discovered that the location of the charged particle and the mutual attraction of the nearby particles cause the position update of the candidate solutions. AEFA has good exploitation ability with limited global search capacity due to the strong ability of interaction of the particles^40^. AEFA solely uses the electrostatic force. This technique of updating population only draws other agents to the lead agent quickly by limiting population variety. As a result, AEFA is susceptible to becoming temporarily trapped.

**Figure 1:**
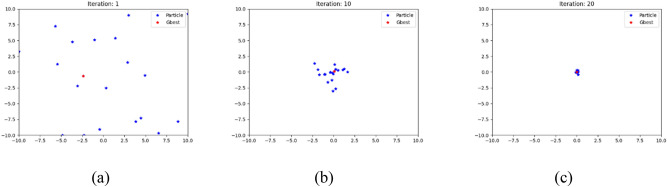
Distribution of 20 agents in AEFA with (**a**) the random distribution of agents, (**b**) the updated locations of agents after 10 iterations and (**c**) the gathered positions of agents.

Figure 1 displays the migration of 20 agents utilizing the Sphere function, with the dimension set to 2 for visualization. The upper and lower bounds set to 10, − 10 and agents are shown at various iterations. The lead agent leads the migration as the other agents begin to transverse the solution space at iteration one. At iteration 10, AEFA starts to collect agents around the ideal area in the problem space as informed by the lead agent. The strong attraction force discourage exploration in other particles. This leads to reduction in population diversity as seen in Fig. 1. Peradventure the lead agent is trapped in some local optimal, other agents will also become trapped. In other words, the lead agent dominates the exploring capabilities of AEFA. The other particles need to be more capable of exploration and exploitation by weakening the leadership of lead agent. Also, the lead agent requires a local optimum avoidance approach. This drawback serves as the foundation for this work's motive.

#### Cuckoo search nest replacement strategy

Cuckoo Search (CS) nest replacement operator is used to replace some nests randomly with newly produced solutions to enhance the algorithm's exploration capability. With the use of this replacement operator in the CS, the exploration capability of the algorithm is quite strong^52–54^. Due to the efficiency that CS has, this method is applied to AEFA as an aid tool for its poor exploration ability.

This process involves by replacing a set of nests with new values based on a probabilistic selection. It is possible to choose any nest ${Y}_{i} \left(i\in \left[1,\dots ,N\right]\right)$ with a probability of ${p}_{a}\in [\mathrm{0,1}]$. A uniform random number $\mathrm{R}$ within [0, 1] is assigned in order to carry out this procedure. When $\mathrm{R}$ falls below ${p}_{a}$, the nest ${Y}_{i}$ is chosen and adjusted as shown in Eq. (15). In all other respects, ${Y}_{i}$ is unchanged. The Eq. (15) is shown as follows,

15 $${Y}_{i}\left(t+1\right)=\left\{\begin{array}{ll}{Y}_{i}+rand\cdot \left({Y}_{y}-{Y}_{j}\right),& \text{with probability }{p}_{a},\\ {Y}_{i },& \text{with probability }\left(1-{p}_{a}\right),\end{array}\right.$$

where $y$ and $j$ are random numbers from 1 to $N$ and *rand* is a random number with normal distribution.

#### Refraction learning

Refraction Learning (RL) is based on the idea that light rays bend when they pass through an air-to-water transition. As an object's medium shifts the velocity shifts as well, bending in the direction of the boundary's normal. This theory aims to assist a candidate’s solutions in leaving the sub-optimal while retaining variety^55^. This kind of opposition-based learning can be considered more advanced to avoid sub-optimality. Refraction learning is used in Whale Optimization Algorithm (WOA) and Equalized Grey Wolf Optimizer (EGWO)^24,56^. In both of the applications, it can be seen from the statistical results that the local optimality is avoided via RL method. RL equations are stated as follows,

16 $${x}^{{^{\prime}}*}=(\mathrm{LB}+\mathrm{UB})/2+(\mathrm{LB}+\mathrm{UB})/(2k\eta )-{x}^{*}/(k\eta )$$

where ${x}^{*}$ represents a variable in the potential solution and $\eta$ is the specified refraction index which is expressed as follows,

17 $$\eta =\frac{{\sin}{\theta }_{1}}{{\sin}{\theta }_{2}}$$

18 $${\sin}{\theta }_{1}=\left((\mathrm{LB}+\mathrm{UB})/2-{x}^{*}\right)/h$$

19 $${\sin}{\theta }_{2}=\left({x}^{{^{\prime}}*}-(\mathrm{LB}+\mathrm{UB})/2\right)/{h}^{{^{\prime}}}$$

**Figure 2:**
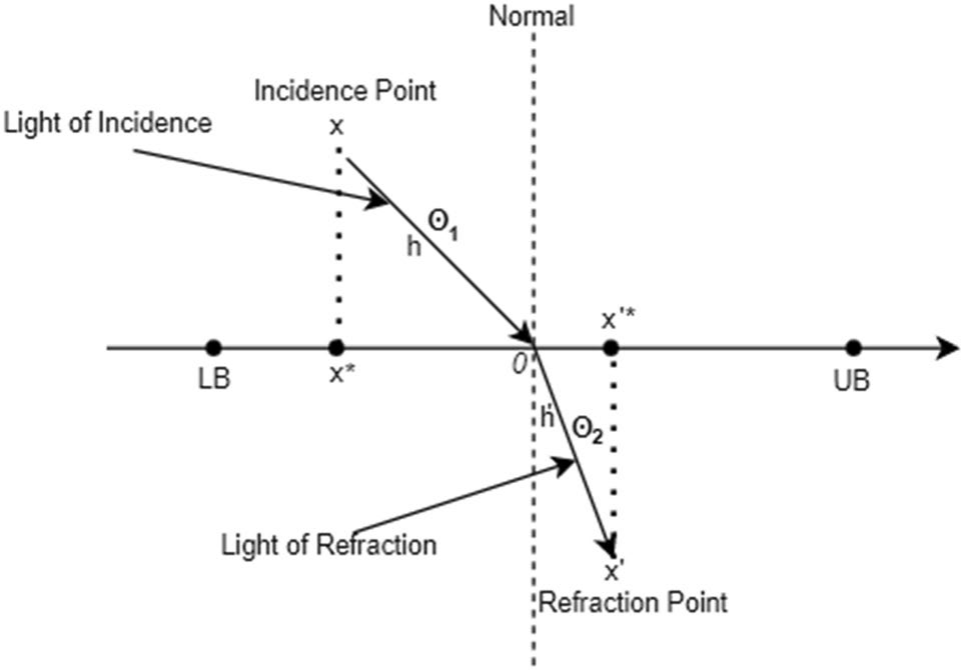
Fundamentals of light refraction.

The refraction absorption index $k$ is expressed as,

20 $$k=h/{h}^{^{\prime}}$$

where Fig. 2 depicts the light's refraction with all variables, $x$ and ${x}^{^{\prime}}$ denote the incidence point and the refraction point, respectively. Serving as upper limit, upper limit and center are the symbol of $\mathrm{LB}$,$\mathrm{UB}, O$. The parameters $h$ and ${h}^{{^{\prime}}}$ define the distances from $x$ to $O$ and from ${x}^{{^{\prime}}}$ to $O$. The refracted solution of ${x}^{*}$ is ${x}^{{^{\prime}}*}$.

## Experimental results

Experiments are carried out on 20 standard benchmark functions to confirm the efficiency of AEFA-CSR for solving global optimization functions. The algorithms; Artificial Electric Field Algorithm (AEFA)^9^, Cuckoo Search (CS)^47^, Differential Evolution (DE)^6^, Firefly Algorithm (FA)^8^, Particle Swarm Optimization (PSO)^4^, Jaya Algorithm (JAYA)^57^, Hybrid-Flash Butterfly Optimization Algorithm (HFBOA)^58^, Sand Cat Swarm Optimization (SCSO)^59^, Salp Swarm Algorithm with Local Escaping Operator (SSALEO)^60^, Transient Search Optimization (TSO)^61^ and Chaotic Hybrid Butterfly Optimization Algorithm with Particle Swarm (HPSOBOA)^62^ were chosen for a detailed observation. The algorithms are chosen in a way to give a better insight to the readers such as well-studied and commonly used ones, recently developed ones which gained attention from the researchers in a short period of time and finally hybrid algorithms that are made up of powerful optimizers. Each algorithm is individually tested on the functions for 30 independent trials to ensure about their problem solving capabilities. The Wilcoxon Rank Sum test and the nonparametric Friedman test are used for statistical testing to represent the variations in the algorithms’ performances^63,64^. Several parameter combinations are put up to examine the influence of each control parameter on each of the algorithms. Additionally, convergence analysis, overall effectiveness with changing populations and dimensions, exploration and exploitation analyses, computational complexity tests are conducted. Afterwards, the efficiency of AEFA-CSR is validated using such real-world engineering problems; optimization of antenna S-parameters, welded-beam and compression designs.

### Benchmark functions

Table 1 displays the fundamental properties of the 20 functions that were chosen for testing. F1 through F7 are unimodal functions that have just one global optimum solution within the specified boundary and are typically used to gauge the algorithm's ability to exploit regions of potential solution. F8–F20 are multimodal functions, F8–F13 are high-dimensional multimodal functions and F14–F20 are fixed-dimensional multimodal functions. These functions have multiple local extremes in each self-defined function's domain which are capable of detecting global exploration and can cause the algorithm's premature convergence.

**Table 1:** Benchmark functions.

No	Function	Dimension	Range	Fmin
${F}_{1}$	${f}_{1}(x)={\sum }_{i=1}^{n} {x}_{i}^{2}$	N	$[-\mathrm{100,100}]$	0
${F}_{2}$	${f}_{2}(x)={\sum }_{\mathrm{i}=1}^{n} \left|{x}_{i}\right|+{\prod }_{i=1}^{n} \left|{x}_{i}\right|$	N	$[-\mathrm{10,10}]$	0
${F}_{3}$	${f}_{3}(x)={\sum }_{i=1}^{n} {\left({\sum }_{j-1}^{i} {x}_{j}\right)}^{2}$	N	$[-\mathrm{100,100}]$	0
${F}_{4}$	${f}_{4}(x)={\mathrm{min}}_{i} \left\{\left|{x}_{i}\right|,1\le i\le n\right\}$	N	$[-\mathrm{100,100}]$	0
$${\begin{array}{c}\\ F\end{array}}_{5}$$	${f}_{5}(x)=\sum_{i=1}^{d} \sum_{j=1}^{i} {x}_{j}^{2}$	N	$[-\mathrm{65.536,65.536}]$	0
${F}_{6}$	${f}_{6}(x)={\sum }_{i=1}^{n} {\left(\left[{x}_{i}+0.5\right]\right)}^{2}$	N	$[-\mathrm{100,100}]$	0
${F}_{7}$	${f}_{7}(x)={\sum }_{i=1}^{n} i{x}_{i}^{4}+\mathrm{random}[\mathrm{0,1})$	N	$[-\mathrm{1.28,1.28}]$	0
${F}_{8}$	${f}_{8}(x)=1-\mathrm{cos}\left(2\pi \sqrt{\sum_{i=1}^{d} {x}_{i}^{2}}\right)+0.1\sqrt{\sum_{i=1}^{d} {x}_{i}^{2}}$	N	$[-\mathrm{100,100}]$	0
${F}_{9}$	${f}_{9}(x)={\sum }_{i=1}^{n} \left[{x}_{i}^{2}-10\mathrm{cos}\left(2\pi {x}_{i}\right)+10\right]$	N	$[-\mathrm{5.12,5.12}]$	0
${F}_{10}$	${f}_{10}(x)=-20\mathrm{exp}\left(-0.2\sqrt{\frac{1}{n}{\sum }_{i=1}^{n} {x}_{i}^{2}}\right)-\mathrm{exp}\left((1/n){\sum }_{i=1}^{n} \mathrm{cos}\left(2\pi {x}_{i}\right)\right)+20+e$	N	$[-\mathrm{32,32}]$	0
${F}_{11}$	${f}_{11}(x)=1/4000{\sum }_{i=1}^{n} \sum {x}_{i}^{2}-{\prod }_{i=1}^{n} \mathrm{cos}\left({x}_{i}/\sqrt{i}\right)+1$	N	$[-\mathrm{600,600}]$	0
${F}_{12}$	${f}_{12}(x)=\pi /n\left\{{\sum }_{i=1}^{n-1} {\left({y}_{i}-1\right)}^{2}\left[1+10{\mathrm{sin}}^{2}\left(\pi {y}_{i+1}\right)\right]+{\left({y}_{n}-1\right)}^{2}\right\}+{\sum }_{i=1}^{n} u\left({x}_{i},\mathrm{10,100,4}\right)+\pi /n10\mathrm{sin}\left(\pi {y}_{1}\right)$ $${y}_{i}=1+{x}_{i}+(1/4)u\left({x}_{i},a,k,m\right)=\left\{\begin{array}{ll}k{\left({x}_{i}-a\right)}^{m}& {x}_{i}>a\\ 0& -a<{x}_{i}<a\\ k{\left(-{x}_{i}-a\right)}^{m}& {x}_{i}<-a\end{array}\right.$$	N	$[-\mathrm{50,50}]$	0
${F}_{13}$	${f}_{13}(x)=0.1\left\{{\sum }_{i=1}^{n} {\left({x}_{i}-1\right)}^{2}\left[1+{\mathrm{sin}}^{2}\left(3\pi {x}_{i}+1\right)\right]+{\left({x}_{n}-1\right)}^{2}\left[1+{\mathrm{sin}}^{2}\left(2\pi {x}_{n}\right)\right]\right\}+0.1{\mathrm{sin}}^{2}\left(3\pi {x}_{1}\right)+{\sum }_{i=1}^{n} u\left({x}_{i},\mathrm{5,100,4}\right)$	N	$[-\mathrm{50,50}]$	0
${F}_{14}$	${f}_{14}(x)=\left|{x}^{2}+{y}^{2}+xy\right|+|\mathrm{sin}(x)|+|\mathrm{cos}(y)|$	2	$[-\mathrm{500,500}]$	1
${F}_{15}$	${f}_{15}(x)={\mathrm{sin}}^{2}(3\pi x)+(x-1{)}^{2}\left(1+{\mathrm{sin}}^{2}(3\pi y)\right)+(y-1{)}^{2}\left(1+{\mathrm{sin}}^{2}(2\pi y)\right)$	4	$[-\mathrm{10,10}]$	0
${F}_{16}$	${f}_{16}(x)=4{x}_{1}^{2}-2.1{x}_{1}^{4}+1/3{x}_{1}^{6}+{x}_{1}{x}_{2}-4{x}_{2}^{2}+4{x}_{2}^{4}$	2	$[-\mathrm{5,5}]$	− 1.0316
${F}_{17}$	${f}_{17}(x)={\left({x}_{2}-5.1/4{\pi }^{2}{x}_{1}^{2}+5/\pi {x}_{1}-6\right)}^{2}+10(1-(1/8\pi ))\mathrm{cos}{x}_{1}+10$	2	$[-\mathrm{5,5}]$	0.398
${F}_{18}$	${f}_{18}(x)=\left[1+{\left({x}_{1}+{x}_{2}+1\right)}^{2}\left(19-14{x}_{1}+3{x}_{1}^{2}-14{x}_{2}+6{x}_{1}{x}_{2}+3{x}_{2}^{2}\right)\right]\times \left[30+{\left(2{x}_{1}-3{x}_{2}\right)}^{2}\times \left(18-32{x}_{1}+12{x}_{1}^{2}+48{x}_{2}-36{x}_{1}{x}_{2}+27{x}_{2}^{2}\right)\right]$	2	$[-\mathrm{2,2}]$	3
${F}_{19}$	${f}_{19}(x)=-{\sum }_{i=1}^{4} {c}_{i}\mathrm{exp}\left[-{\sum }_{j=1}^{3} {a}_{ij}{\left({x}_{j}-{p}_{ij}\right)}^{2}\right]$	3	$[\mathrm{1,3}]$	− 3.86
${F}_{20}$	${f}_{20}(x)={x}^{2}+2{y}^{2}-0.3\mathrm{cos}(3\pi x)\mathrm{cos}(4\pi y)+0.3$	2	$[-\mathrm{100,100}]$	0

### Parameters

The values published in the original publications or often used in many research are chosen as parameters for the corresponding algorithms which are provided in Table 2.

**Table 2:** Parameter settings.

Algorithms	Parameter setting
AEFA	Coulombs constant ${k}_{0}=500$, $\gamma =30$
CS	Probability to regenerate nest $Pa$ $=0.25$, Mutation rate $r$ $=0.05$
DE	Mutation factor $F$ $=0.5,$ Crossover ratio $CR$ $=0.7$
FA	Light absorption coefficient $\zeta =1$, step size $s=0.2$
PSO	${w}_{\mathrm{max}}=0.9, {w}_{\mathrm{min}}=0.2, {c}_{1}={c}_{2}=2,{v}_{\mathrm{max}=}6$
AEFA-CSR	Refraction absorption $k=1000, \mathrm{Refraction index }\eta =1000,$ Probability replacing bad particle $Pa$ $=0.1$, Coulombs constant ${k}_{0}=500$,$\gamma =30$
HFBOA	Power exponent $a=0.1$ Switch parameter $p=0.6$ Chaotic factor $\mu =4$ initial value for attractiveness ${\beta }_{0}=1,{\alpha }_{0}=0.2$ sensory modality ${c}_{0}=0.35$
SCSO	Roulette wheel selection [0, 360], $C$ $=0.35,$ $SM$ $=2$
SSALEO	C1 = $[2/\mathrm{e},2]$
TSO	$k=2, z\in [\mathrm{0,2}]$
HPSOBOA	$$\begin{array}{c}{a}_{\text{first }}=0.1,{a}_{\text{final }}=0.3,c(0)=0.01,p=0.6\\ x(0)=0.315,\rho =0.295,{c}_{1}={c}_{2}=0.5\end{array}$$

### Overall effectiveness

In this study, the results from Tables 3, 4, 5, 6 and 7 were used to evaluate the Overall Effectiveness (OE) of the AEFA-CSR to that of its counterparts. Equation (21) demonstrates that the number of test functions and losses for each algorithm can be used to determine the OE of the comparison algorithms^60^.

21 $$OE=\left(\frac{N-L}{N}\right)\times 100$$

where N is the total number of function and L is number the number of losses incurred by an algorithm. In the tables, W and T indicate the number of wins and the number of ties respectively.

**Table 3:** F1–F20 comparison with dimension = *30* and population = *30.*

		AEFA-CSR	AEFA	CS	DE	FA	PSO	JAYA	HFBOA	SSALEO	TSO	HPSOBOA	SCSO
F1	Avg	**8.70E−55**	6.92E−24	6.24E−3	3.95E−13	2.63E−3	4.44E−8	2.21E−13	7.06E−19	1.11E−8	3.31E−18	3.00E−9	1.31E−34
	Std	**2.00E−56**	3.77E−24	3.28E−3	3.63E−13	6.35E−4	1.57E−8	1.45E−13	3.09E−20	1.82E−9	3.00E−18	7.76E−10	8.38E−38
F2	Avg	**1.29E−28**	1.06E+2	1.71E−1	1.12E−7	3.91	8.45	1.68E−9	7.73E+12	2.11E−4	5.13E−8	4.49E−1	9.52E−22
	Std	**2.17E−30**	3.07E+1	5.92E−2	6.58E−8	7.46E−1	4.82	6.15E−10	2.42E+11	5.63E−5	5.66E−7	4.63E−2	2.09E−23
F3	Avg	**9.52E−48**	2.09E+3	3.47E+2	8.53E+3	9.39E+1	1.42E+1	6.58E+3	4.16E−19	8.64E−6	5.76E−8	9.61E−8	4.78E−17
	Std	**6.59E−49**	6.81E+2	1.15E+2	3.43E+3	6.77E+1	4.77	1.70E+4	2.45E−20	1.74E−6	8.07E−28	4.65E−8	6.21E−18
F4	Avg	**9.55E−27**	1.60	5.35	8.57	1.11E−1	6.42E−1	1.02E+1	2.63E−12	5.99E−4	8.72E−11	5.72E−6	3.33E−10
	Std	**2.30E−27**	9.41E−1	1.56	5.34	3.79E−2	1.71E−1	7.06	4.30E−15	5.42E−4	6.52E−15	2.28E−6	3.72E−13
F5	Avg	**1.44E−54**	6.86	3.73E−2	2.30E−12	8.04E−1	2.75E−8	2.73E−13	4.59E−17	1.11E−6	1.32E−22	1.94E−7	2.65E−28
	Std	3.32E−55	2.37E−1	1.91E−2	2.50E−12	3.79E−1	7.87E−9	1.11E−13	2.46E−18	6.39E−7	**0**	2.72E−7	3.76E−28
F6	Avg	**0**	2.03	6.30E−3	4.43E−13	2.28E−3	2.87E−9	3.45	5.00	1.07E−8	2.84E−3	6.28	6.08
	Std	**0**	5.00E−1	3.44E−3	5.10E−13	6.41E−4	2.61E−9	6.71E−1	7.96E−2	6.36E−10	1.12E−3	1.02	2.87
F7	Avg	1.31E−3	1.33	4.41E−2	1.84E−2	5.42E−1	5.61	2.31E−2	**8.99E−5**	5.24E−4	3.98E−4	2.96E−4	1.31E−4
	Std	1.00E−4	3.87E−2	1.58E−2	5.73E−3	1.76E−1	3.58	1.37E−2	**2.13E−5**	2.50E−4	1.36E−4	1.25E−4	9.12E−5
F8	Avg	**8.59E−19**	1.36	1.42	2.78E−1	5.63E−1	4.09E−1	5.45E−1	1.16E−16	3.74E−2	1.77E−12	4.93E−6	2.70E−18
	Std	**4.39E−19**	3.47E−1	2.28E−1	4.49E−2	1.32E−1	6.07E−2	4.91E−1	5.15E−20	8.37E−7	6.39E−17	4.17E−6	4.61E−19
F9	Avg	**0**	3.94E+1	7.84E+1	1.58E+2	8.23E+1	9.02E+1	6.61E+1	**0**	5.49E−9	7.57E−15	2.21E−6	**0**
	Std	**0**	1.44E+1	1.40E+1	1.20E+1	2.68E+1	2.85E+1	2.96E+1	**0**	3.76E−10	0	5.26E−7	**0**
F10	Avg	**4.44E−16**	2.66E−1	1.36	2.70E−7	1.79E−1	5.14E−5	5.31E−7	1.43E−13	2.39E−5	2.66E−10	2.60E−5	3.87E−15
	Std	**0**	2.08E−13	7.49E−1	1.46E−7	1.53E−3	1.88E−5	1.03E−7	3.76E−13	6.08E−6	6.57E−14	1.79E−5	**0**
F11	Avg	**0**	4.87E−1	8.66E−2	8.21E−5	1.22E−2	1.01E−2	8.92E−3	**0**	4.20E−8	**0**	1.34E−10	**0**
	Std	**0**	4.58E−1	5.33E−2	4.04E−9	8.00E−3	1.42E−3	5.44E−5	**0**	5.90E−10	**0**	8.70E−11	**0**
F12	Avg	**4.26E−24**	2.17	1.30	2.78E−11	1.25E−5	1.03E−2	5.26E−1	4.72E−1	8.20E−11	4.71E−5	9.48E−1	9.77E−1
	Std	**1.99E−25**	1.04	6.18E−1	4.30E−13	5.09E−6	9.35E−10	4.05E−1	1.11E−1	7.27E−12	1.52E−5	3.94E−2	9.87E−2
F13	Avg	5.44E−3	7.41	6.81E−1	**2.73E−7**	4.55E−3	4.02E−3	1.39E+3	2.58	7.57E−3	4.47E−5	2.89	2.85
	Std	5.69E−4	6.81	1.21	**9.77E−11**	4.51E−3	2.68E−9	2.64E+2	7.89E−3	4.31E−4	1.02E−6	2.94E−1	7.72E−2
F14	Avg	**1.00**	**1.00**	**1.00**	**1.00**	**1.00**	**1.00**	9.90	**1.00**	**1.00**	**1.00**	**1.00**	**1.00**
	Std	**0**	3.42E−5	**0**	**0**	1.05E−4	**0**	1.59	**0**	5.00E−7	**0**	3.50E−6	**0**
F15	Avg	**1.35E−31**	**4.01E−27**	**1.35E−31**	**1.35E−31**	7.54E−9	1.35E−31	3.94E−5	1.22E−4	5.68E−14	1.88E−4	2.26E−1	5.63E−2
	Std	**0**	4.42E−27	6.68E−47	6.68E−47	5.44E−9	6.68E−47	5.16E−5	6.60E−5	3.65E−14	3.11E−4	4.66E−2	3.17E−3
F16	Avg	**− 1.03**	**− 1.03**	**− 1.03**	**− 1.03**	**− 1.03**	**− 1.03**	− 1.02	− 9.41E−1	**− 1.03**	− 4.37E−1	− 7.94E−1	− 1.02
	Std	**0**	4.51E−16	4.51E−16	4.51E−16	4.51E−16	4.51E−16	1.74E−2	5.76E−2	**0**	3.81E−2	3.38E−2	1.02E−3
F17	Avg	**3.97E−1**	**3.97E−1**	**3.97E−1**	**3.97E−1**	**3.97E−1**	**3.97E−1**	**3.97E−1**	3.98E−1	3**.97E−1**	8.44E−1	4.13E−1	4.06E−1
	Std	**0**	5.64E−17	5.64E−17	5.64E−17	5.64E−17	5.64E−17	1.61E−4	1.84E−4	**0**	2.08E−5	1.03E−1	8.44E−3
F18	Avg	**3.00**	**3.00**	**3.00**	**3.00**	**3.00**	**3.00**	3.62	3.00	**3.00**	2.59E+1	5.22	3.03
	Std	**0**	1.22E−2	**0**	**0**	**0**	**0**	2.99	7.26E−4	**0**	1.30E+1	2.63	1.96E−2
F19	Avg	**− 3.86**	− 3.62	**− 3.86**	**− 3.86**	**− 3.86**	**− 3.86**	− 3.65	− 3.23	**− 3.86**	− 3.45	− 3.25	− 3.27
	Std	**0**	3.45E−1	1.80E−15	1.80E−15	1.80E−15	2.40E−3	1.15E−1	3.25E−1	**0**	2.21E−1	2.60E−1	1.47E−1
F20	Avg	**0**	**0**	**0**	**0**	2.20E−6	**0**	1.22	**0**	1.12E−11	**0**	2.48E−9	**0**
	Std	**0**	**0**	**0**	**0**	2.24E−6	**0**	1.53E−1	3.52E−13	**0**	5.90E−9	**0**	**0**
W/L/T		9/2/9	0/17/3	0/13/7	1/12/7	0/16/4	0/14/6	0/20/0	1/15/4	0/16/4	0/18/2	0/20/0	0/16/4
OE		**90%**	15%	35%	40%	20%	30%	0%	25%	20%	10%	0%	20%

Significant values are in bold.

**Table 4:** F1–F13 comparison with dimension = *50* and population = *30.*

		AEFA-CSR	AEFA	CS	DE	FA	PSO	JAYA	HFBOA	SSALEO	TSO	HPSOBOA	SCSCO
F1	Avg	**1.85E−54**	1.37E+1	2.61	7.20E−6	1.29E−2	1.44E−3	3.11E−7	1.31E−15	4.29E−8	8.96E−27	6.09E−9	1.66E−12
	Std	**1.88E−55**	1.92E−1	7.48E−1	4.85E−6	3.07E−3	1.70E−3	1.15E−6	1.31E−17	8.63E−9	7.76E−28	5.10E−9	2.05E−13
F2	Avg	**6.75E−28**	2.23E+2	2.18	1.07E−3	4.56E+1	2.64E+1	1.15E−6	7.40E+23	5.86E−4	8.31E−11	1.91E+23	1.71E−18
	Std	**1.54E−28**	3.88E+1	1.63	5.95E−4	3.50E+1	1.51E+1	1.07E−6	1.32E+23	5.52E−4	1.20E−14	1.51E+21	2.65E−20
F3	Avg	**5.92E−46**	4.78E+3	3.84E+3	6.99E+4	1.45E+4	5.58E+2	6.08E+4	1.61E−17	5.67E−5	2.75E−14	2.96E−7	4.11E−25
	Std	**3.05E−48**	1.12E+3	7.48E+2	9.93E+3	5.76E+3	1.65E+2	1.33E+4	1.20E−18	3.76E−6	1.50E−18	2.59E−7	4.28E−25
F4	Avg	**4.75E−26**	8.42	1.41E+1	5.32E+1	3.96	2.34	5.07E+1	1.78E−12	1.02E−3	9.25E−13	8.25E−6	1.64E−13
	Std	**2.78E−26**	2.22	2.33	2.03E+1	3.45	3.39E−1	1.04E+1	2.96E−18	1.67E−4	8.13E−13	3.81E−8	8.90E−15
F5	Avg	**5.08E−52**	8.47E+2	2.44E+1	5.13E−5	1.40E+2	1.85E−2	1.03E−6	1.82E−17	4.58E−6	4.00E−18	5.52E−7	8.91E−22
	Std	6.08E−55	6.67E+2	9.87	4.52E−5	2.47E+1	1.49E−2	1.47E−8	1.99E−18	2.72E−6	1.61E−21	1.66E−9	**0**
F6	Avg	**0**	9.42E+1	2.93	7.53E−6	1.34E−2	1.29E−3	7.76	9.76	4.77E−8	5.07E−3	1.13E+1	1.10E+1
	Std	**0**	6.17E+1	1.13	6.97E−6	3.36E−3	3.14E−3	6.05E−1	2.94E−1	8.16E−9	2.48E−3	2.53E−1	6.34E−2
F7	Avg	1.32E−3	4.13E+2	1.63E−1	6.48E−2	1.01	3.09E+1	5.26E−2	**1.16E−4**	5.51E−4	3.85E−4	3.84E−4	2.02E−4
	Std	2.66E−4	4.99E+1	4.56E−2	2.31E−2	3.03E−1	2.29E+1	3.53E−2	3.46E−5	**1.08E−5**	1.09E−4	6.23E−5	4.27E−5
F8	Avg	**7.56E−19**	3.16	3.58	7.45E−1	1.22	6.66E−1	7.77E−1	1.81E−14	2.07E−2	4.34E−11	7.61E−6	7.02E−14
	Std	2.18E−18	5.67E−1	4.63E−1	9.66E−2	1.52E−1	7.88E−2	2.84E−1	7.46E−17	2.89E−5	1.65E−13	2.04E−6	**1.52E−20**
F9	Avg	**0**	1.66E+2	1.68E+2	3.58E+2	1.72E+2	2.75E+2	1.40E+2	**0**	2.50E−8	**0**	7.99E−6	**0**
	Std	**0**	4.41E+1	1.81E+1	2.30E+1	3.47E+1	4.39E+1	5.06E+1	**0**	3.18E−9	**0**	1.99E−5	**0**
F10	Avg	**4.44E−16**	3.57	3.49	1.19	4.88E−1	6.00E−1	7.29E−5	1.88E−13	3.79E−5	1.00E−8	2.40E−5	1.86E−15
	Std	**0**	1.39	6.50E−1	1.45E−4	1.98E−2	6.44E−1	3.56E−5	**0**	2.41E−6	**0**	5.39E−5	**0**
F11	Avg	**0**	7.76	9.45E−1	1.41E−3	2.20E−2	4.34E−3	4.60E−3	**0**	2.14E−7	2.36E−15	1.13E−10	**0**
	Std	**0**	2.11	8.85E−2	4.05E−3	5.75E−3	5.54E−3	1.99E−2	**0**	5.13E−9	**0**	1.10E−10	**0**
F12	Avg	6.43E−3	6.20	3.70	4.23E+1	4.87E−1	1.86E−2	1.28	7.22E−1	**2.42E−10**	3.47E−5	1.09	1.07
	Std	5.18E−4	1.93	8.71E−1	1.05E−2	3.09E−3	2.79E−4	6.06E−1	1.74E−2	**4.07E−11**	5.31E−6	9.19E−2	1.14E−3
F13	Avg	4.45E−2	5.28E+2	3.23E+1	9.36	5.50E−3	8.48E−3	1.52E+3	4.87	3.80E−1	**9.23E−5**	4.88	4.88
	Std	3.86E−4	4.73	1.32E+1	7.81E−1	3.17E−4	1.18E−4	1.21	2.39E−3	1.41E−3	**6.26E−5**	1.12E−2	4.76E−2
W/L/T		8/3/2	0/13/0	0/13/0	0/13/0	0/13/0	0/13/0	0/13/0	1/10/2	1/12/0	1/11/2	0/13/0	0/11/2
OE		**76.93%**	0%	0%	0%	0%	0%	0%	23.07%	7.69%	15%	0%	15.38%

Significant values are in bold.

**Table 5:** F1–F13 comparison with dimension = *50* and population = *60.*

		AEFA-CSR	AEFA	CS	DE	FA	PSO	JAYA	HFBOA	SSALEO	TSO	HPSOBOA	SCSCO
F1	Avg	**3.65E−43**	1.72E−3	4.86	1.20E−2	1.43E−2	2.06E−5	1.99E−9	1.34E−21	2.83E−8	1.80E−21	6.47E−9	4.69E−36
	Std	**2.35E−43**	8.62E−4	1.40	4.28E−3	3.67E−3	1.37E−6	2.34E−10	1.13E−22	4.31E−9	1.24E−22	1.62E−9	2.06E−42
F2	Avg	**2.17E−21**	9.25E+1	9.18	1.18E−1	4.73E+1	2.00E+1	2.19E−7	1.89E+21	2.58E−4	1.33E−8	8.19E+21	5.12E−11
	Std	**7.29E−22**	3.19E+1	2.74	4.80E−2	4.58E+1	4.58	1.09E−7	1.72E+20	1.53E−4	5.72E−10	2.65E+21	4.43E−12
F3	Avg	2.31E−24	2.24E+3	7.16E+3	9.22E+4	4.13E+3	3.29E+2	5.68E+4	8.33E−20	8.46E−6	3.13E−14	7.02E−7	**2.30E−28**
	Std	2.10E−24	4.00E+1	9.25E+2	9.38E+3	2.01E+3	8.59E+1	2.09E+3	2.55E−22	8.11E−6	1.32E−14	9.34E−8	**5.64E−29**
F4	Avg	**6.26E−20**	1.61	1.12E+1	7.92E+1	1.06	1.85	4.45E+1	4.78E−17	3.81E−4	5.48E−11	4.43E−6	1.94E−10
	Std	2.87E−20	9.88E−1	9.76E−1	7.71	1.74E−2	1.55E−1	3.94	**2.76E−21**	2.40E−4	1.51E−16	2.59E−6	2.02E−15
F5	Avg	**5.68E−42**	6.88E+1	3.98E+1	8.87E−2	1.12E+2	1.41E−4	2.90E−8	1.70E−23	1.13E−6	2.48E−19	5.91E−7	1.22E−16
	Std	**3.76E−42**	2.75E+1	1.06E+1	3.30E−2	3.30E+1	5.39E−5	2.51E−8	1.25E−30	9.01E−7	1.06E−24	5.71E−7	3.04E−41
F6	Avg	**0**	3.73	4.63	1.34E−2	1.25E−2	2.70E−5	7.75	9.61	2.84E−8	7.06E−4	1.09E+1	1.05E+1
	Std	**0**	3.00	1.16	4.68E−3	3.70E−4	4.55E−6	2.56E−1	1.25E−1	4.96E−9	1.04E−4	3.50E−1	3.75E−1
F7	Avg	1.49E−3	7.96E+1	9.06E−2	7.58E−2	5.27E−1	3.06E+1	3.07E−2	**3.12E−5**	3.74E−4	2.50E−4	4.60E−4	8.70E−5
	Std	1.10E−3	1.01E+1	1.54E−2	1.67E−2	1.53E−1	1.86E+1	6.26E−3	2.32E−5	2.04E−4	9.53E−5	1.42E−4	**1.75E−5**
F8	Avg	1.05E−13	1.42	3.12	1.31	8.99E−1	5.93E−1	7.18E−1	**2.01E−15**	1.18E−2	3.54E−10	3.80E−6	9.57E−14
	Std	1.32E−14	2.01E−1	2.65E−1	1.20E−1	5.00E−2	8.16E−2	1.47E−1	3.31E−18	2.44E−3	2.60E−15	1.17E−6	**7.89E−24**
F9	Avg	**0**	6.41E+1	1.74E+2	3.75E+2	1.61E+2	2.31E+2	1.05E+2	**0**	1.43E−8	**0**	5.84E−6	**0**
	Std	**0**	2.48	1.78E+1	1.55E+1	4.97	3.20E+1	4.21	**0**	2.20E−9	**0**	4.39E−7	**0**
F10	Avg	**4.44E−16**	2.32E−1	3.96	4.05E−1	3.62E−1	1.29E−1	1.02E−5	1.71E−13	3.04E−5	4.39E−7	3.81E−5	7.99E−16
	Std	9.86E−32	2.42E−3	3.45E−1	9.68E−3	3.38E−1	1.18E−3	3.72E−7	**0**	2.20E−7	**0**	7.66E−7	**0**
F11	Avg	**0**	1.90	1.03	2.03E−2	2.22E−2	3.12E−3	1.98E−2	**0**	7.98E−8	6.49E−15	4.21E−11	**0**
	Std	**0**	7.10E−1	1.82E−2	4.93E−3	3.23E−3	2.90E−8	6.36E−5	**0**	2.33E−8	0	2.47E−11	**0**
F12	Avg	4.14E−3	1.36	2.46	1.80E−1	8.36E−2	6.22E−3	9.84E−1	6.76E−1	**7.91E−11**	9.60E−6	1.02	1.04
	Std	1.55E−2	8.02E−2	4.52E−1	1.57E−1	7.16E−6	4.94E−9	4.29E−2	9.45E−2	**5.86E−12**	1.26E−6	8.28E−2	4.97E−2
F13	Avg	6.64E−3	1.59E+1	1.07E+1	2.77E−1	6.46E−3	6.23E−3	6.99E+1	4.71	6.67E−1	**3.51E−5**	4.93	4.87
	Std	5.48E−3	1.04	4.79	8.57E−2	3.54E−4	5.22E−6	3.66	2.76E−3	5.49E−3	**2.24E−5**	3.20E−3	2.80E−2
W/L/T		6/5/2	0/13/0	0/13/0	0/13/0	0/13/0	0/13/0	0/13/0	2/9/2	1/12/0	1/11/001	0/13/0	1/10/(2)
OE		**61.53%**	0%	0%	0%	0%	0%	0%	30.76%	7.69%	15.38%	0%	23.07%

Significant values are in bold.

**Table 6:** F1–F13 comparison with dimension = *50* and population = *90.*

		AEFA-CSR	AEFA	CS	DE	FA	PSO	JAYA	HFBOA	SSALEO	TSO	HPSOBOA	SCSCO
F1	Avg	**1.34E−41**	6.44E−25	9.59	2.25E−1	1.39E−2	9.05E−7	6.81E−10	1.13E−24	2.15E−8	3.26E−22	6.12E−9	2.07E−12
	Std	**8.16E−42**	2.74E−26	1.86	9.44E−2	8.58E−4	3.52E−7	9.81E−11	1.56E−30	4.48E−9	9.83E−31	8.70E−10	4.68E−13
F2	Avg	**1.09E−20**	3.87E+1	1.98E+1	6.22E−1	2.31E+1	1.60E+1	8.96E−8	3.14E+20	1.58E−4	7.65E−11	4.07E+21	2.48E−16
	Std	**4.22E−21**	3.45E+1	4.63	9.90E−2	1.10E+1	5.30E−4	9.03E−9	2.30E+17	3.91E−5	2.42E−13	5.49E+20	4.55E+21
F3	Avg	2.19E−24	1.33E+3	9.27E+3	9.69E+4	2.80E+3	2.16E+2	6.20E+4	2.22E−20	2.79E−6	3.26E−20	5.02E−7	**1.10E−28**
	Std	7.31E−25	6.68E+1	1.05E+3	6.61E+3	1.45E+2	4.75	1.54E+4	3.79E−23	1.89E−6	5.56E−24	2.51E−7	**4.27E−33**
F4	Avg	**5.64E−19**	2.28E−1	1.42E+1	8.59E+1	5.97E−1	1.49	3.68E+1	4.90E−17	2.63E−4	6.95E−13	6.16E−6	1.03E−11
	Std	**7.55E−20**	1.99E−2	9.53E−1	2.33	1.62E−1	3.00E−1	1.32E+1	4.43E−20	1.26E−4	2.12E−18	5.70E−7	1.93E−19
F5	Avg	**1.32E−40**	2.07E+1	8.50E+1	1.44	8.28E+1	4.25E−5	3.75E−9	5.94E−23	5.46E−7	8.35E−18	7.41E−7	1.47E−29
	Std	**3.61E−42**	7.62E−2	1.99E+1	4.66E−1	3.76E+1	1.24E−5	7.38E−10	3.69E−30	5.59E−8	3.65E−29	1.41E−8	7.32E−31
F6	Avg	**0**	3.00E−1	8.80	1.94E−1	1.36E−2	6.43E−7	7.82	9.42	2.34E−8	3.24E−4	1.08E+1	1.02E+1
	Std	**0**	0	1.79	3.73E−2	2.50E−3	4.16E−7	4.49E−1	1.16E−1	1.94E−9	3.72E−6	5.40E−1	3.88E−1
F7	Avg	1.30E−3	6.87	9.22E−2	9.46E−2	3.81E−1	2.75E+1	3.40E−2	**2.29E−5**	2.41E−4	1.51E−4	5.26E−4	4.60E−5
	Std	4.00E−4	2.11E−1	1.84E−2	1.46E−2	4.87E−4	9.38	7.00E−3	**6.98E−6**	5.15E−5	3.36E−4	5.85E−5	4.35E−5
F8	Avg	1.14E−13	9.22E−1	3.53	1.73	7.46E−1	5.66E−1	6.40E−1	**1.66E−17**	1.00E−2	5.17E−8	5.29E−6	3.73E−16
	Std	2.94E−16	4.89E−2	2.50E−1	9.34E−2	5.00E−2	**0**	2.00E−1	1.26E−17	3.09E−7	5.98E−17	4.67E−6	7.37E−21
F9	Avg	**0**	3.37E+1	1.82E+2	3.80E+2	1.51E+2	2.19E+2	9.29E+1	**0**	1.16E−8	3.79E−15	1.25E−5	0
	Std	**0**	1.99E+1	1.68E+1	1.33E+1	1.89E+1	4.10E+1	4.57E+1	**0**	5.03E−10	0	5.37E−9	0
F10	Avg	**4.44E−16**	1.86E−4	5.33	9.70E−1	6.70E−1	2.53E−3	6.12E−6	**4.44E−16**	2.62E−5	8.63E−8	4.39E−5	1.71E−11
	Std	**0**	7.10E−15	4.87E−1	1.39E−1	3.73E−3	4.34E−4	5.69E−6	**0**	3.73E−6	**0**	2.20E−5	**0**
F11	Avg	**0**	9.87E−1	1.08	2.47E−1	2.01E−2	4.59E−3	7.97E−3	**0**	5.28E−8	1.48E−8	8.25E−11	**0**
	Std	**0**	2.66E−2	1.68E−2	1.69E−2	6.98E−3	1.00E−8	1.03E−4	**0**	2.71E−9	**0**	2.93E−12	**0**
F12	Avg	6.22E−3	4.07E−1	2.68	1.94	1.20E−2	5.78E−8	8.31E−1	6.42E−1	**5.25E−11**	4.83E−6	9.23E−1	9.86E−1
	Std	0	1.67E−1	3.87E−1	3.99E−1	8.04E−6	1.32E−8	3.22E−1	6.17E−2	1.51E−12	3.79E−10	4.55E−2	5.27E−2
F13	Avg	3.66E−4	3.82	1.22E+1	5.26	5.74E−3	3.66E−3	7.13	4.57	1.00	**9.73E−6**	4.93	4.84
	Std	2.26E−5	2.00	2.43	9.10E−1	6.09E−5	2.50E−6	2.54	3.19E−2	5.49E−3	**2.38E−7**	1.90E−4	3.25E−2
W/L/T		5/5/3	0/13/0	0/13/0	0/13/0	0/13/0	0/13/0	0/13/0	2/8/3	1/12/0	1/12/0	0/13/0	1/10/2
OE		**61.53%**	0%	0%	0%	0%	0%	0%	38.46%	7.69%	7.69%	0%	23.07%

Significant values are in bold.

**Table 7:** F1–F13 comparison with dimension = *100* and population = *30*.

		AEFA-CSR	AEFA	CS	DE	FA	PSO	JAYA	HFBOA	SSALEO	TSO	HPSOBOA	SCSCO
F1	Avg	**4.25E−52**	1.07E+3	3.58E+2	6.85	2.03E−1	2.69	4.15E−3	1.98E−17	5.50E−7	2.65E−12	1.27E−8	8.90E−23
	Std	**1.14E−53**	2.81E+2	8.21E+1	2.45E−1	4.35E−2	1.51	4.08E−4	2.46E−26	2.55E−8	3.00E−18	7.91E−10	1.71E−24
F2	Avg	**1.05E−27**	4.23E+2	1.65E+1	1.24	3.64E+2	1.10E+2	1.83E−3	1.42E+50	1.86E−3	2.82E−9	2.30E+50	2.30E−11
	Std	**1.32E−28**	4.81E+1	2.78	2.62E−2	3.49E+2	2.77E+1	2.84E−4	2.26E+46	1.76E−4	3.00E−18	7.79E+48	2.14E−11
F3	Avg	**1.49E−43**	1.78E+4	3.08E+4	3.26E+5	1.78E+5	1.12E+4	3.05E+5	8.46E−18	4.83E−4	3.32E−17	2.34E−6	1.07E−21
	Std	**9.06E−45**	3.14E+3	4.63E+3	3.63E+2	4.82E+4	2.60E+3	9.12E+4	7.01E−20	2.83E−3	3.00E−18	1.82E−7	9.27E−23
F4	Avg	**1.17E−25**	1.66E+1	2.15E+1	9.59E+1	4.63E+1	8.66	9.14E+1	2.19E−13	1.47E−3	8.44E−11	8.32E−6	5.51E−11
	Std	**6.53E−26**	1.60	1.42	8.56E−1	6.27	1.03	5.82E−1	5.02E−17	3.04E−3	9.63E−14	4.13E−6	1.61E−15
F5	Avg	**8.17E−53**	3.27E+4	6.39E+3	5.69E+1	4.98E+3	1.87E+2	9.12E−2	7.70E−15	4.48E−5	1.22E−16	4.18E−6	4.13E−16
	Std	**2.25E−53**	8.14E+3	1.59E+3	1.41	3.61E+3	1.14E+2	2.63E−2	1.77E−16	1.27E−5	1.00E−16	1.82E−6	1.12E−15
F6	Avg	**0**	1.54E+3	3.54E+2	6.83	2.04E−1	3.54	2.29E+1	2.24E+1	7.07E−7	1.71E−2	2.38E+1	2.35E+1
	Std	**0**	6.19E+2	9.03E+1	4.65E−1	4.43E−2	1.80	1.10	1.04	2.10E−7	9.61E−3	3.82E−1	1.18
F7	Avg	2.02E−3	1.89E+3	1.30	5.99E−1	2.51	2.99E+2	3.85E−1	**1.74E−4**	6.32E−4	5.88E−4	7.60E−4	2.19E−4
	Std	1.98E−3	1.74E+2	3.53E−1	1.39E−1	6.25E−1	1.41E+2	1.19E−1	**8.74E−5**	8.80E−4	2.43E−4	2.97E−4	1.34E−4
F8	Avg	**1.22E−18**	7.40	8.70	3.37	4.74	1.42	2.62	2.99E−15	4.21E−2	3.61E−12	1.08E−5	4.35E−14
	Std	**1.51E−19**	7.81E−1	7.76E−1	5.25E−3	5.45E−1	1.19E−1	3.35E−1	5.13E−18	5.95E−3	5.64E−15	1.29E−5	6.55E−16
F9	Avg	**0**	8.42E+2	4.27E+2	8.79E+2	4.91E+2	7.13E+2	4.08E+2	**0**	2.52E−7	**0**	1.22E−5	**0**
	Std	**0**	1.26E+2	3.52E+1	3.04E+1	5.75E+1	6.52E+1	4.74E+1	**0**	1.43E−8	**0**	4.77E−7	**0**
F10	Avg	**4.44E−16**	7.81	6.83	1.72	1.54	2.63	6.78E−1	1.09E−13	8.54E−5	8.56E−13	2.54E−5	2.33E−15
	Std	**0**	7.82E−1	6.13E−1	1.71	2.10E−1	2.52E−1	2.52E−3	3.55E−15	1.28E−6	**0**	1.28E−5	**0**
F11	Avg	**0**	4.99E+1	4.49	7.76E−1	9.33E−2	4.30E−2	5.75E−2	**0**	3.28E−6	**0**	2.62E−10	**0**
	Std	**0**	9.76	9.60E−1	5.14E−3	1.88E−2	1.73E−2	2.10E−2	**0**	7.16E−6	**0**	4.49E−11	**0**
F12	Avg	2.95E−2	1.55E+2	2.54E+1	2.94E+4	6.32	1.87	1.97E+4	9.35E−1	1.12E−3	**5.71E−5**	1.12	1.14
	Std	5.61E−3	4.00E+2	9.18E−1	1.64E+3	1.64	9.16E−1	2.95	1.56E−3	1.07E−3	**1.63E−4**	2.95E−2	1.29E−2
F13	Avg	2.65	2.37E+5	6.60E+3	3.67E+4	1.79E+1	1.52E+1	1.82E+5	9.98	6.52	**1.60E−4**	9.93	9.90
	Std	2.93E−1	1.95E+4	2.61E+2	2.24E+4	1.64E+1	9.21	1.06E+5	3.11E−4	1.86	**5.65E−6**	1.88E−4	8.45E−3
W/L/T		8/3/2	0/13/0	0/13/0	0/13/0	0/13/0	0/13/0	0/13/0	1/10/2	0/13/0	2/9/2	0/13/0	0/11/2
OE		**76.9%**	0%	0%	0%	0%	0%	0%	23.07%	0%	30.7%	0%	15.38%

Significant values are in bold.

### Dimension analysis

Given that dimensionality has a substantial impact on optimization accuracy, F1 through F13 are expanded from 30 to 100 dimensions to test the algorithms' abilities in solving the problems. The outputs of each algorithm is then assessed. The mean value (*Avg*) and standard deviation (*Std*) are used as the assessment indices to give the experiments greater credibility. *Avg* might indicate the algorithm's quality and accuracy of the solutions, while *Std* indicates the algorithm's stability. Population size is 30 and the maximum number of iterations for all algorithms is set to 1000.

The experimental findings with a dimension of *30* are displayed in Table 3. The analysis suggests that with unimodal functions (F1–F7), AEFA-CSR finds the best near ideal solution. It is noticeable that AEFA-CSR outperforms the other algorithms by a larger margin. This is because of the included RL mechanism which improves the algorithm's ability for local search as well as exploitation. With F8–F20 which are multimodal functions, AEFA-CSR performs the best on F8–F12, F14–F20 and obtained the global optimum for F9 and F11. All of the outcomes produced by AEFA-CSR are superior to those that are produced by AEFA. This suggests that after incorporating CS approach the improved population variety, the algorithm's exploration ability has increased in comparison to AEFA. A solid balance between exploitation and exploration is also successfully achieved by the algorithm as observed in the results of the fixed dimension functions.

Tables 3, 4 and 7 show the experimental study for varying dimensions while the population size kept as constant 30. Tables 4, 5 and 6 show the experimental study for varying population size while the dimension kept as constant 50. It can be observed that for a fixed population size, when the problem size grows in all the cases AEFA-CSR is superior to other compared algorithms in terms of Overall Effectiveness (OE) ranging from 76.93 to 90.0%. Similarly, it is apparent that for a fixed dimension size, when the population size grows, AEFA-CSR produced higher OE values ranging from 61.53 to 76.93%.

### Convergence analysis

**Figure 3:**
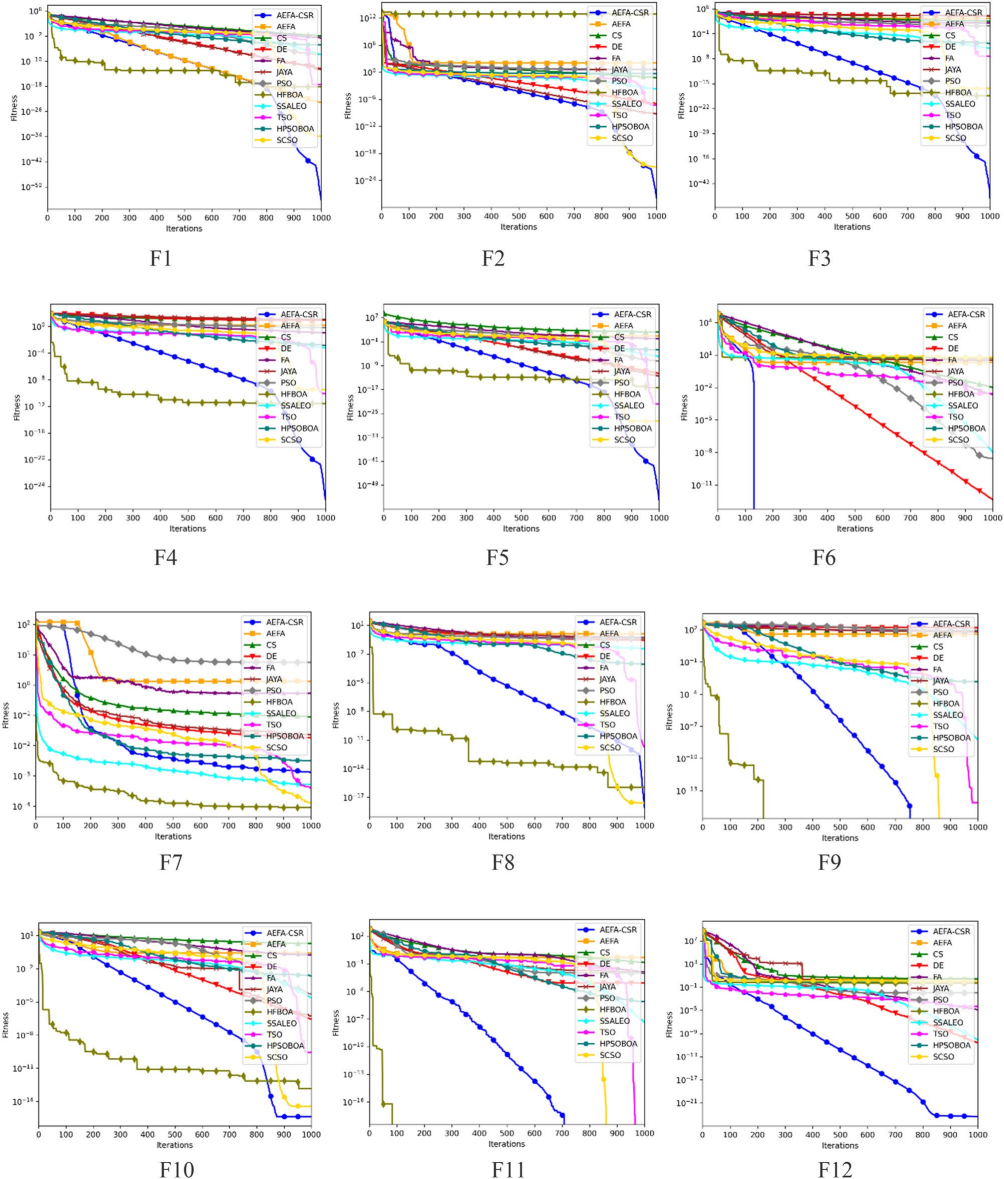

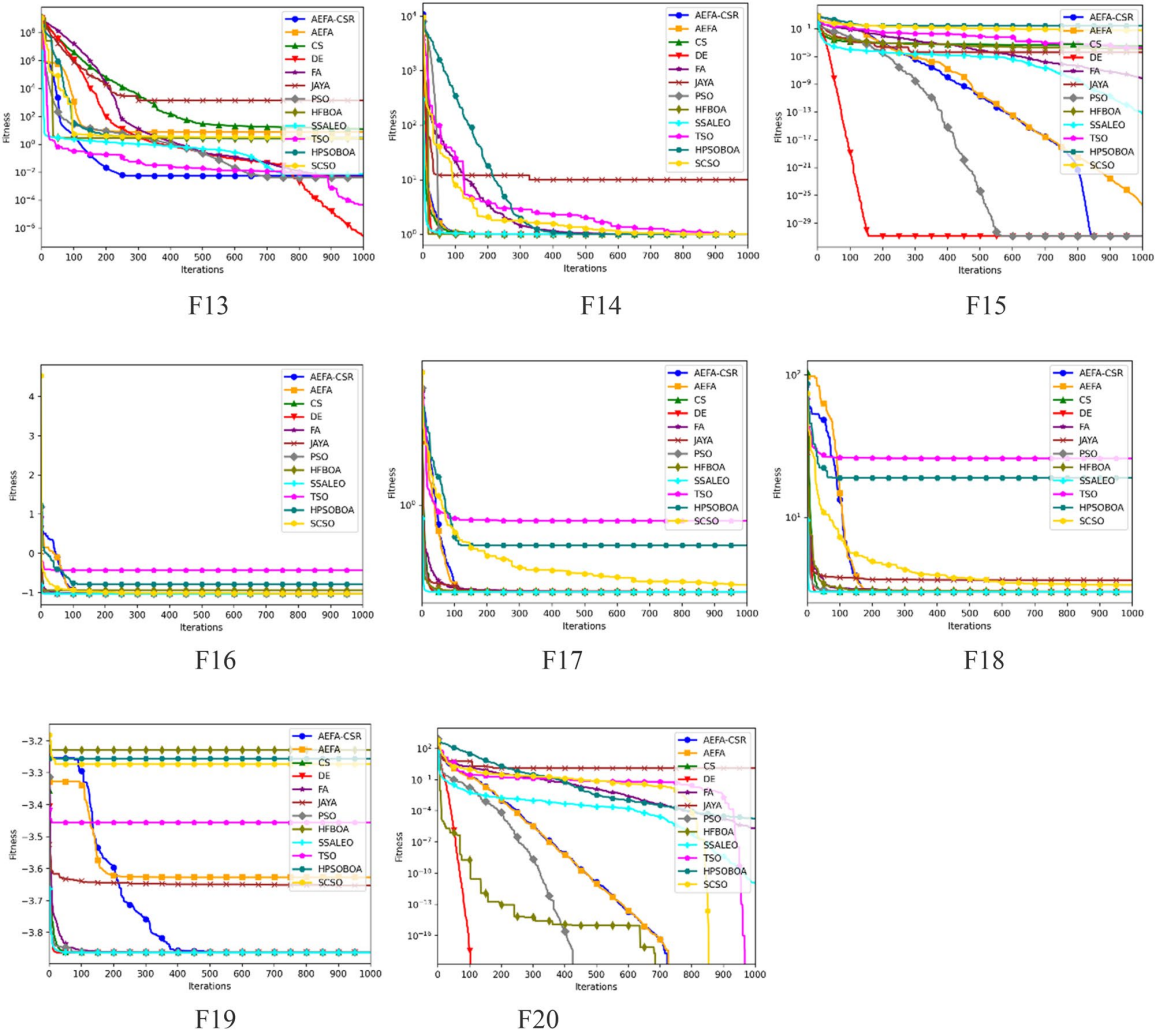
Convergence trajectory with dimension = *30*.

The convergence trajectories of 12 algorithms are presented in Fig. 3 to further analyze how well different algorithms accomplish convergence while addressing optimization functions. The dimension for the functions; F1–F13 is set to 30, while the functions F14–F20 are the fixed dimension functions. It is evident that the convergence precision of AEFA-CSR is significantly better on unimodal functions; F1–F6. AEFA-CSR maintained an extraordinary convergence rate and converges to the global optimal on F9, F11, F14, F16 and F20 in it performance with multimodal functions; F8–F20.

The convergence efficiency of AEFA-CSR is noticeably better than the AEFA. It is demonstrated that the population diversification adjustments and the introduction of RL technique are quite successful. The experimental findings show that AEFA-CSR has improved its optimization capability and convergence performance. Worthy of note is the algorithm’s rapid convergence will be applicable in optimization problems that the convergence is the essential component.

### Statistical test

Garcia et al. made the point that it is insufficient to compare metaheuristic algorithm performance using only mean and standard deviation^65^. Therefore, during the iterative process, inescapable factors that have an impact on the experimental outcomes have been added^66,67^. The Wilcoxon Rank Sum test and the Friedman test are used in this study to examine the effectiveness of the algorithms.

The Friedman test is used to evaluate the experiment's validity by comparing the proposed AEFA-CSR to other algorithms. The Friedman test, one of the most popular and commonly applied statistical tests which is used to find significant differences between the outputs of two or more algorithms^68^. Table 8 displays the results of the Friedman tests. The algorithm with the lowest ranking is thought to be the most effective algorithm according to the Friedman test findings. The suggested AEFA-CSR is always rated first in the various scenarios; *30*, *50* and *100* dimensions with population set to 30 according to the results in the Table 8. The AEFA-CSR has stronger competitive edge over the other algorithms.

**Table 8:** Friedman’s test for dimension *30*, *50* and *100* dimensions for 20 functions.

Test	Dim	AEFA-CSR	AEFA	CS	DE	FA	PSO	JAYA	HFBOA	SSALEO	TSO	HPSOBOA	SCSCO
Friedman Value	30	2.48	8.48	7.55	5.45	7.55	6.53	8.50	5.88	6.18	5.75	8.15	5.53
Friedman Rank		1	10	8	2	8	7	11	5	6	4	9	3
Friedman Value	50	1.96	10.85	9.62	8.85	8.38	7.46	8.23	4.42	5.08	3.19	6.31	3.65
Friedman Rank		1	12	11	10	9	7	8	4	5	2	6	3
Friedman Value	100	1.92	11.00	10.00	9.83	8.67	8.00	8.58	3.33	4.83	2.92	5.50	3.42
Friedman Rank		1	12	11	10	9	7	8	3	5	2	6	4

The significance threshold *p* for the Wilcoxon Rank Sum test is set at 0.05. The technique is shown to be statistically better when *p* < 0.05. Table 9 displays the results achieved by Wilcoxon Rank Sum test. The symbols +/−/= denote that the suggested ways are better, worse or equal to than the existing approach^67^. Table 9 demonstrates that AEFA-CSR consistently offers R + values that are greater than R- values. Additionally, AEFA-CSR is superior than the other algorithms as an observation from Table 9 which shows that *p* values of the six algorithms are less than 0.05. That is an indication of which imply they are substantially different from AEFA-CSR. Table 8 further reveals when the dimension expanded from *30* to *100* with population set to *30*, the + value of AEFA-CSR increased. This indicates the performance of AEFA-CSR does not decline like other algorithms. It is able to produce substantial improvement compared to the other algorithms as dimension increase. The findings demonstrate that the suggested AEFA-CSR has higher level of solution accuracy.

**Table 9:** Wilcoxon Rank sum test *30*, *50* and *100* dimensions for 20 functions.

Dim	AEFA-CSR vs	−	+	=	R−	R+	p-value
30	AEFA	0	17	3	0	153	2.93E−04
	CS	0	13	7	0	91	1.47E−03
	DE	1	12	7	8	83	8.78E−03
	FA	1	15	4	5	131	1.12E−03
	PSO	1	13	6	6	99	3.51E−03
	JAYA	0	20	0	0	210	8.90E−05
	HFBOA	1	15	4	9	127	2.28E−03
	SSALEO	1	17	2	15	157	1.85E−03
	TSO	2	15	3	24	129	1.29E−02
	HPSOBOA	1	18	1	10	180	6.25E−04
	SCSO	1	15	4	8	128	1.92E−03
50	AEFA	0	13	0	0	91	1.47E−03
	CS	0	13	0	0	91	1.47E−03
	DE	0	13	0	0	91	1.47E−03
	FA	1	12	0	4	87	3.73E−03
	PSO	1	12	0	6	85	5.77E−03
	JAYA	0	13	0	0	91	1.47E−03
	HFBOA	1	10	2	7	59	2.08E−02
	SSALEO	2	11	0	20	71	7.47E−02
	TSO	3	9	1	32	46	5.83E−01
	HPSOBOA	1	12	0	9	82	1.07E−02
	SCSO	1	10	2	8	58	2.62E−02
100	AEFA	0	13	0	0	91	1.47E−03
	CS	0	13	0	0	91	1.47E−03
	DE	0	13	0	0	91	1.47E−03
	FA	0	13	0	0	91	1.47E−03
	PSO	0	13	0	0	91	1.47E−03
	JAYA	0	13	0	0	91	1.47E−03
	HFBOA	1	9	2	7	48	3.67E−02
	SSALEO	2	11	0	19	72	6.40E−02
	TSO	3	8	2	29	37	7.22E−01
	HPSOBOA	1	12	0	9	69	1.86E−02
	SCSO	1	10	2	8	58	2.62E−02

Conclusively, AEFA-CSR is more competitive than both traditional algorithms such as DE, PSO and innovative algorithms such as FA, JAYA, CS, SCSO and hybrid algorithms such as HPSOBOA. The newly introduced strategies are to be credited for the proposed algorithm’s greater achievements. Due to the RL solution strategy, improves local optimal escape mechanism and the CS lessen the dominance of the lead agent. Therefore, combining the two approaches significantly enhance the ability of AEFA to solve multimodal and unimodal functions.

### Sensitivity analysis to parameters

Assessment to the sensitivity of parameters is also carried out in this part to investigate the impact of various parameters of AEFA-CSR. Population size, iteration number and dimension are maintained at 30, 1000 and 30 throughout the experiment. The starting values of the $k$, $\eta$ in Eqs. (15) and (16) are set to 1 and 0.25, then are altered throughout the test. The parameter $Pa$ is varied within the range [0, 1] and the author of CS suggests a value of 0.25, while parameters $k$ and $\eta$ is varied within [1, 1000]^47^. As indicated in Table 10, there are eight variations of the AEFA-CSR developed. Each of which represents a combination of various parameters. Note that these settings can be changed to fit the particular problem. It can be also noted that for $k$, $\eta$ and $Pa$, the values of 1000, 1000 and 0.1 are used in earlier experiments. The variation of AEFA-CSR with these parameters performed best based on the Friedman rank.

**Table 10:** Statistical Test for parameter combination with dimension = *30*.

		$k$=$\eta$=1 pa = 0.25	$k$=$\eta$=10 pa = 0.25	$k$=$\eta$=100 pa = 0.25	$k$=$\eta$=1000 pa = 0.25	$k$=$\eta$=1000 pa = 0.1	$k$=$\eta$=1000 pa = 0.15	$k$=$\eta$=1000 pa = 0.2	$k$=$\eta$=1000 pa = 0.3
F1	Avg	3.67E−34	**3.22E−55**	2.51E−54	9.99E−53	8.70E−55	1.22E−54	7.62E−54	8.61E−54
	Std	2.30E−35	**1.75E−56**	4.34E−56	1.97E−55	2.00E−56	8.01E−56	9.89E−56	8.24E−54
F2	Avg	7.06E−17	7.01E−28	6.48E−28	2.69E−28	**1.29E−28**	2.95E−28	2.42E−28	4.42E−28
	Std	5.51E−18	5.32E−29	8.97E−29	6.91E−30	**2.17E−30**	4.34E−29	1.19E−28	1.58E−28
F3	Avg	9.40E−5	1.30E−44	1.06E−46	4.77E−46	**9.52E−48**	8.39E−47	1.66E−46	2.35E−44
	Std	2.39E−8	1.93E−49	3.08E−48	**9.49E−50**	6.59E−49	2.20E−48	2.10E−48	2.35E−47
F4	Avg	1.06E−15	1.27E−26	1.80E−26	4.40E−26	**9.55E−27**	1.63E−26	2.33E−26	2.40E−25
	Std	6.55E−16	**1.77E−27**	1.45E−26	1.90E−26	2.30E−27	5.69E−27	2.76E−27	1.40E−26
F5	Avg	2.99E−33	1.10E−53	2.46E−54	1.06E−51	**1.44E−54**	1.85E−54	6.30E−54	6.43E−53
	Std	2.99E−34	2.15E−54	2.33E−54	3.80E−53	3.32E−55	7.54E−56	1.19E−54	2.97E−55
F6	Avg	3.33E−2	**0**	**0**	**0**	**0**	**0**	**0**	**0**
	Std	**0**	**0**	**0**	**0**	**0**	**0**	**0**	**0**
F7	Avg	9.59E−3	1.80E−3	1.34E−3	2.05E−3	**1.31E−3**	1.86E−3	1.93E−3	1.61E−3
	Std	2.00E−3	8.75E−4	1.64E−4	1.51E−3	1.00E−4	1.03E−4	**6.84E−5**	1.41E−3
F8	Avg	1.19E−1	5.14E−1	7.01E−19	1.33E−18	8.59E−19	1.73E−18	6.50E−19	**6.06E−19**
	Std	6.29E−6	4.22E−2	5.10E−19	2.89E−19	4.39E−19	**1.43E−19**	4.03E−19	2.62E−19
F9	Avg	3.35E+00	7.51E−1	**0**	**0**	**0**	**0**	**0**	**0**
	Std	2.42E+00	4.97E−1	**0**	**0**	**0**	**0**	**0**	**0**
F10	Avg	6.22E−14	**4.44E−16**	**4.44E−16**	**4.44E−16**	**4.44E−16**	**4.44E−16**	**4.44E−16**	**4.44E−16**
	Std	2.84E−14	**0**	**0**	**0**	**0**	**0**	**0**	**0**
F11	Avg	6.09E−7	**0**	**0**	**0**	**0**	**0**	**0**	**0**
	Std	**0**	**0**	**0**	**0**	**0**	**0**	**0**	**0**
F12	Avg	2.80E−2	1.03E−2	6.91E−3	1.43E−23	**4.26E−24**	6.91E−3	3.45E−3	3.48E−3
	Std	4.26E−3	3.03E−4	6.84E−4	2.50E−24	**1.99E−25**	1.03E−4	5.18E−4	8.34E−4
F13	Avg	1.83E−3	2.19E−3	**7.32E−4**	3.66E−3	5.44E−3	3.29E−3	4.71E−3	4.71E−3
	Std	3.29E−4	4.29E−4	**8.49E−5**	5.49E−4	5.69E−4	2.19E−4	5.17E−4	2.74E−4
F14	Avg	**1**	**1**	**1**	**1**	**1**	**1**	**1**	**1**
	Std	**0**	**0**	**0**	**0**	**0**	**0**	**0**	**0**
F15	Avg	**1.35E−31**	**1.35E−31**	**1.35E−31**	**1.35E−31**	**1.35E−31**	**1.35E−31**	**1.35E−31**	**1.35E−31**
	Std	**0**	**0**	**0**	**0**	**0**	**0**	**0**	**0**
F16	Avg	**-1.03**	**-1.03**	**-1.03**	**-1.03**	**-1.03**	**-1.03**	**-1.03**	**-1.03**
	Std	**0**	**0**	**0**	**0**	**0**	**0**	**0**	**0**
F17	Avg	**3.97E−1**	**3.97E−1**	**3.97E−1**	**3.97E−1**	**3.97E−1**	**3.97E−1**	**3.97E−1**	**3.97E−1**
	Std	**0**	**0**	**0**	**0**	**0**	**0**	**0**	**0**
F18	Avg	**3**	**3**	**3**	**3**	**3**	**3**	**3**	**3**
	Std	0	0	0	0	0	0	0	0
F19	Avg	− 3.65	− 3.73	− **3.86**	− **3.86**	− **3.86**	− **3.86**	− **3.86**	− **3.86**
	Std	**0**	**0**	**0**	**0**	**0**	**2.06E−2**	**0**	**0**
F20	Avg	**0**	**0**	**0**	**0**	**0**	**0**	**0**	**0**
	Std	**0**	**0**	**0**	**0**	**0**	**0**	**0**	**0**
Friedman Value		6.60	4.80	3.88	4.63	3.28	4.13	4.15	4.55
Friedman Rank		8	7	2	6	1	3	4	5

Significant values are in bold.

As seen in Table 10, AEFA-CSR with the parameters set as $k$ = 1, $\eta$ = 1 and $Pa$ = 0.25 finds poorer results on all of the test functions compared to when the parameters of AEFA-CSR is set as $k$ = 1000, $\eta$ = 1000 and $Pa$ = 0.1 with the exception of F1, F13 and F14-F20 where results are comparable, this imply that in functions with high dimensions (F1-F13)$, k$ = 1000, $\eta$ = 1000 and $Pa$ = 0.1 is more robust to handle them with more accuracy. Also, result from Table 10 shows that with the parameters $k$ = 1000, $\eta$ = 1000 and $Pa$ = 0.25 AEFA-CSR performance is comparable to, when the parameters are set as $k$ = 10, $\eta$ = 10 and $Pa$ = 0.25 and $k$ = 100, $\eta$ = 100 and $Pa$ = 0.25 with the exception of F8, F9 and F12 where the performance of $k$ = 10, $\eta$ = 10 and $Pa$ = 0.25. Additionally, the result of varying $Pa$ from 0.1 to 0.3 keeping $k$ and $\eta$ at 1000 have no significant difference on the extraordinary performance of the AEFA-CSR. From Table 11, it can be seen that there is no significant difference between the best set of parameters which is $k$ = $\eta$  = 1000 and Pa = 0.1 against $k$ = $\eta$  = 100 and Pa = 0.25, is $k$ = $\eta$  = 1000 and Pa = 0.25, $k$ = $\eta$  = 1000 and Pa = 0.2 and is $k$ = $\eta$  = 1000 and Pa = 0.3 as depicted by the P-values.

**Table 11:** Wilcoxon Rank Sum test for parameter combination.

$k$ = $\eta$ = 1000; Pa = 0.1 vs	−	+	=	R−	R+	P-value
$k$ = $\eta$ = 1; Pa = 0.25	1	13	6	8	97	5.213E−3
$k$ = $\eta$ = 10; Pa = 0.25	2	9	9	8	58	2.623E−2
$k$ = $\eta$ = 100; Pa = 0.25	2	7	11	14	31	3.139E−1
$k$ = $\eta$ = 1000; Pa = 0.25	1	8	11	9	36	1.097E−1
$k$ = $\eta$ = 1000; Pa = 0.15	1	9	10	9	46	5.934E−2
$k$ = $\eta$ = 1000; Pa = 0.2	2	7	11	14	31	3.139E−1
$k$ = $\eta$ = 1000; Pa = 0.3	2	7	11	14	31	3.139E−1

**Figure 4:**
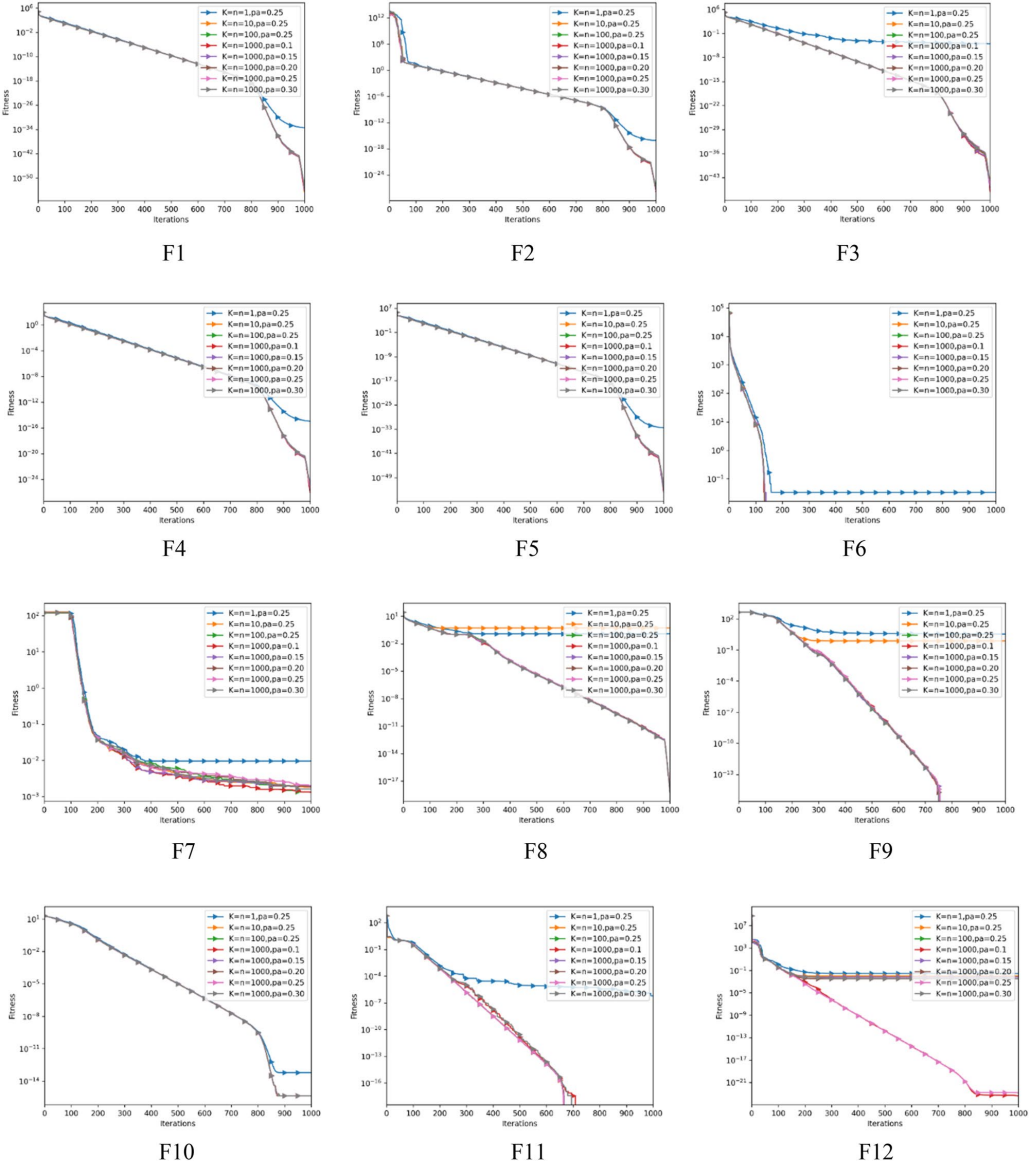

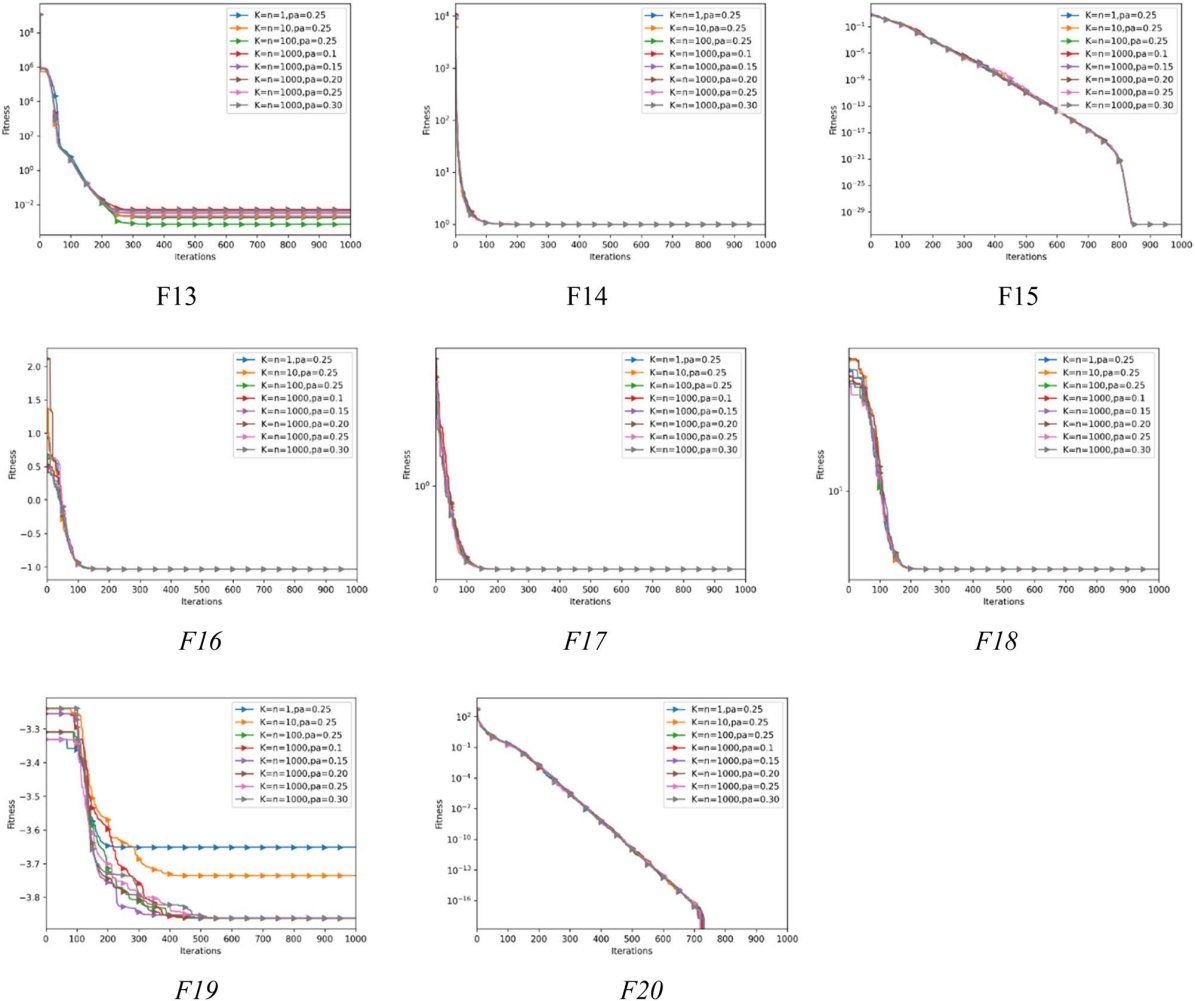
Convergence trajectory values for different parameter combinations.

The average objective function values are illustrated for all test functions with number of independent runs as 30 are shown in Fig. 4 for various combinations of the $k$, $\eta$, and $Pa$. As shown in Fig. 4, the parameter combination obtained show comparable exceptional outcomes for the majority of test functions when $k$ and $\eta$ = 1000, incases pairings of the $k$ and $\eta$ = 1, and 10. AEFA-CSR tends to perform poorly compared to when $k$ and $\eta$ = 1000. The illustrated convergence trajectory depict that to attain the best performance for AEFA-CSR, $k$ and $\eta$ may be tuned to a somewhat big number ideally 1000.

### Exploration and exploitation analysis

Exploration is an ability of an optimization algorithm pursuing the diverse solutions in the unexplored area while exploitation is an ability of an optimization algorithm pursuing the solutions around the optimum solution of a problem. Since F1 and F2 are unimodal functions, they are quite appropriate to observe the algorithm’s exploitation ability. Similarly, F10 and F12 are multimodal functions which have multiple local optimums and they are quite suitable for measuring the exploration ability of the algorithm.

When there is an increase in the dimension, the local optimum points in the multimodal functions increase drastically. As it is indicated in Tables 3, 4 and 7, the proposed AEFA-CSR produced better results on varying dimensions 30, 50 and 100. It is a good indication that the algorithm overcome the multiple local optimum points by reaching the global optimum. This is achieved by a balanced exploration and exploitation.

**Figure 5:**
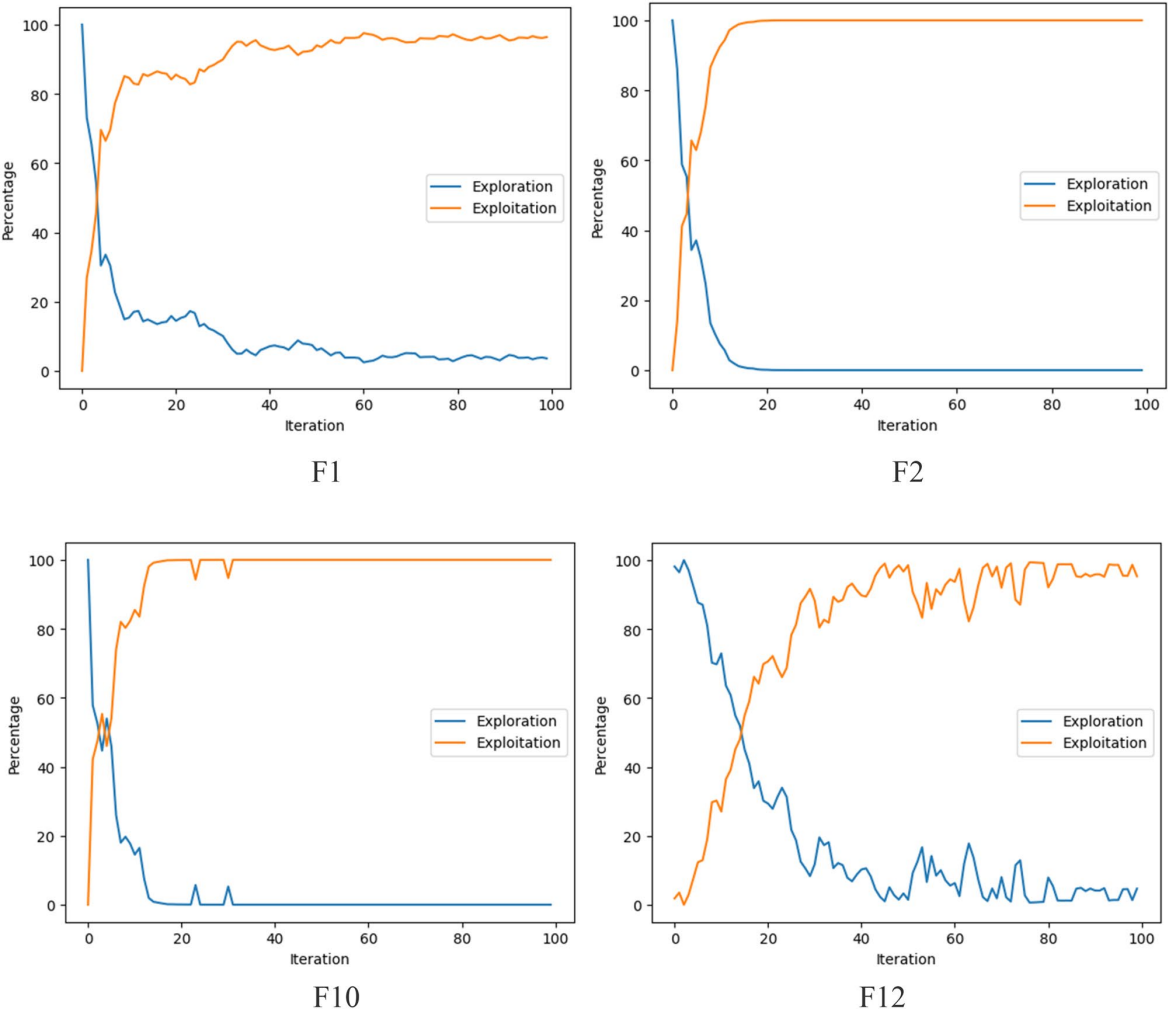
Exploration and exploitation stages through the optimization process.

Additionally, Fig. 5 is given to show the exploration and the exploitation stages of the algorithm AEFA-CSR graphically. For the functions analyzed in this figure, it is seen that the algorithm starts with a broad exploration and narrow exploitation. As the optimization process goes on, the balance between these are assembled.

### Computational complexity

The computational complexity *Big O* notation is one of the metrics to evaluate the performance of metaheuristics. As it is shown in the Algorithm 3, there is only one loop and it is considered as *O* (*N*) where *N* is the number of agents in the population. When it comes to the entire complexity which include modifying the agents towards the optimum solution by calculating the fitness values, it is considered as *O* (${\text{max }}_{{\text{iter}}{\text{ation}}}$ x *N* x *D*) where ${\text{max }}_{\text{iteration}}$ is the number of iterations and *D* is the dimension.

AEFA-CSR is compared with its competitors with respect to their computational time in Table 12. When we have a separate glance on the algorithms AEFA and CS, it is obvious that these algorithms require higher CPU time than the others. Since the proposed algorithm AEFA-CSR is a combination of AEFA, CS and RL, each of the method needs to be carried out during the optimization process individually. Therefore, the CPU time of AEFA-CSR is not always better than the compared methods because of its complex nature. Overall, it can be said that AEFA-CSR requires more computational time, but its efficiency is a way ahead of these algorithms. By considering the significant contributions of the AEFA-CSR, even in real engineering problems, an equilibrium can be constructed between the high accuracy and the amount of time required to solve the problems.

**Table 12:** CPU time comparison for benchmarks at dimension = *30* and population = *30.*

Function	AEFA-CSR	AEFA	CS	DE	FA	PSO	JAYA	HFBOA	SSALEO	TSO	HPSOBOA	SCSO
F1	79.56	22.25	33.05	5.68	34.84	40.20	3.03	5.71	3.68	1.34	3.03	24.87
F2	41.69	26.83	34.26	6.25	35.34	41.35	3.19	6.64	3.84	1.40	3.19	18.21
F3	30.17	92.44	56.38	15.76	44.93	52.50	6.00	27.47	6.55	2.71	6.00	21.30
F4	24.14	64.92	34.37	5.45	23.66	39.89	2.99	5.53	3.70	1.32	2.99	17.73
F5	31.16	26.26	35.31	17.38	13.28	50.73	6.32	31.65	6.75	3.00	6.32	21.02
F6	24.22	23.44	35.11	5.97	10.02	22.59	3.13	6.24	3.77	1.36	3.13	17.94
F7	24.62	24.14	118.38	6.95	10.22	11.48	3.37	7.94	3.66	1.47	3.37	18.06
F8	24.34	24.25	124.78	6.74	10.28	12.04	3.32	7.32	3.92	1.43	3.32	17.92
F9	24.12	24.17	71.91	6.05	9.96	11.28	3.11	6.38	3.47	1.38	3.11	17.73
F10	24.54	24.44	44.46	7.22	10.56	12.27	3.54	7.95	4.03	1.47	3.54	18.01
F11	24.69	24.46	37.42	7.20	10.29	12.40	3.58	7.78	4.09	1.51	3.58	48.35
F12	26.13	25.59	43.12	9.89	11.15	13.19	4.30	13.86	4.83	1.87	4.30	34.13
F13	25.79	25.49	44.46	9.82	11.19	13.02	4.12	12.34	4.66	1.78	4.12	18.51
F14	5.21	4.73	113.29	3.06	10.18	1.08	0.40	5.77	1.16	0.32	0.40	1.72
F15	5.20	4.89	33.17	2.82	10.10	1.07	0.40	5.62	1.15	0.32	0.40	1.72
F16	5.00	4.73	15.22	2.51	10.03	0.95	0.30	5.01	1.06	0.28	0.30	1.60
F17	5.05	4.86	15.76	7.65	29.75	2.91	0.97	15.39	3.25	0.86	0.97	4.91
F18	5.21	4.79	17.25	8.21	30.11	3.11	1.22	16.77	3.42	0.95	1.22	5.13
F19	7.54	6.32	41.40	6.07	10.90	2.36	1.31	11.46	2.02	0.74	1.31	3.15
F20	5.13	4.70	53.21	2.61	10.13	1.01	0.34	5.34	1.12	0.29	0.34	1.69

### Engineering problems application

#### Optimization of antenna S-parameters

The suitability and efficiency of the algorithm in solving engineering problems are shown in this section. In order to demonstrate the suitability of the algorithm, a test suite that is made up of eight test problems for antenna design is chosen. The results are compared to those of other algorithms. Several formulas aimed at analyzing different antenna design problem known as test functions make up the test suite^69,70^. These enable effective assessment of an algorithm's performance.

It is not practical to evaluate optimization algorithms using electromagnetic simulation, since doing so for an antenna often takes a lot of time. Therefore, it is desirable for the pre-test of many antenna optimization techniques to have an effective test suite with analytical test functions. Several authors have proposed several test suites^69–71^. However, the test suite intended for antenna parameter optimization is rarely researched. The features of many antennas types using various types of formulas can be represented by a suitable test suite for antenna design. The objective functions that Zhang et al.^72^ successfully researched and proposed a test suite which covers a diverse characteristic of various types of antenna problems^72,73^ as displayed in Table 13.

**Table 13:** Test functions for antenna S-parameter optimization.

Function types	Formula	Antenna type	F_min_
Unimodal	${F}_{21}({\varvec{x}})=20\mathrm{log}\left(2\left(\sum_{i=1}^{n} \left|{\left(\mathrm{sin}\left(\frac{{x}_{i}}{8}\right)\right)}^{2}\right|+\prod_{i=1}^{n} \left|\mathrm{sin}\left(\frac{{x}_{i}}{8}\right)\right|\right)+1\right)$	Single	0
	${F}_{22}({\varvec{x}})=20\mathrm{log}\left(10{\left(\sum_{i=1}^{n} {x}_{i}^{2}\right)}^{2}+1\right)$	Multiple	0
	${F}_{23}({\varvec{x}})=20\mathrm{log}\left(10{\left(\sum_{i=1}^{n} 0.01{i}^{5}{x}_{i}^{2}\right)}^{2}+1\right)$	Single and multiple	0
	${F}_{24}({\varvec{x}})=20\mathrm{log}\left(\left(\sum_{i=1}^{n-1} \left(100{\left({x}_{i+1}-{x}_{i}^{2}\right)}^{2}-{\left({x}_{i}-1\right)}^{2}\right)\right)+1\right)$	Multiple	0
Multimodal	${F}_{25}({\varvec{x}})=100\sqrt{\left|{x}_{2}+1-0.01{\left({x}_{1}-10\right)}^{2}\right|}+0.01\left|{x}_{1}\right|$	Multiple	0
	${F}_{26}({\varvec{x}})=20\mathrm{log}\left(0.01{\left(\sum_{i=1}^{n} \left|{x}_{i}\right|\right)}^{2}{\left(\mathrm{sin}\left(0.8{x}_{1}\right)+2\right)}^{4}+1\right)$	Multiple	0
Composite	$$\begin{array}{c}{F}_{27}(x)=20log\left(\left(\sum_{i=1}^{n-1} \left(100{\left({x}_{i+1}-{x}_{i}^{2}\right)}^{2}-{\left({x}_{i}-1\right)}^{2}\right)\right)+1\right)\\ +20log\left(0.01{\left(\sum_{i=1}^{n} \left|{x}_{i}\right|\right)}^{2}{\left(\mathrm{sin}\left(0.8{x}_{1}\right)+2\right)}^{4}+1\right)\end{array}$$	Multiple	0
	$$\begin{array}{c}{F}_{28}(x)=100\sqrt{\left|{x}_{2}+1-0.01{\left({x}_{1}-10\right)}^{2}\right|}+0.01\left|{x}_{1}\right|\\ +20log\left(0.01{\left(\sum_{i=1}^{n} \left|{x}_{i}\right|\right)}^{2}{\left(\mathrm{sin}\left(0.8{x}_{1}\right)+2\right)}^{4}+1\right)\end{array}$$	Multiple	0

The dimensions of the test functions F21–F24 and F26–F28 with the exception of F25 which is a non-scalable test function are set to 8 as suggested by Zhang et al.^72^. Parameters of each algorithm as seen Table 2 are maintained with population size of 20 and iteration size of 500.

**Figure 6:**
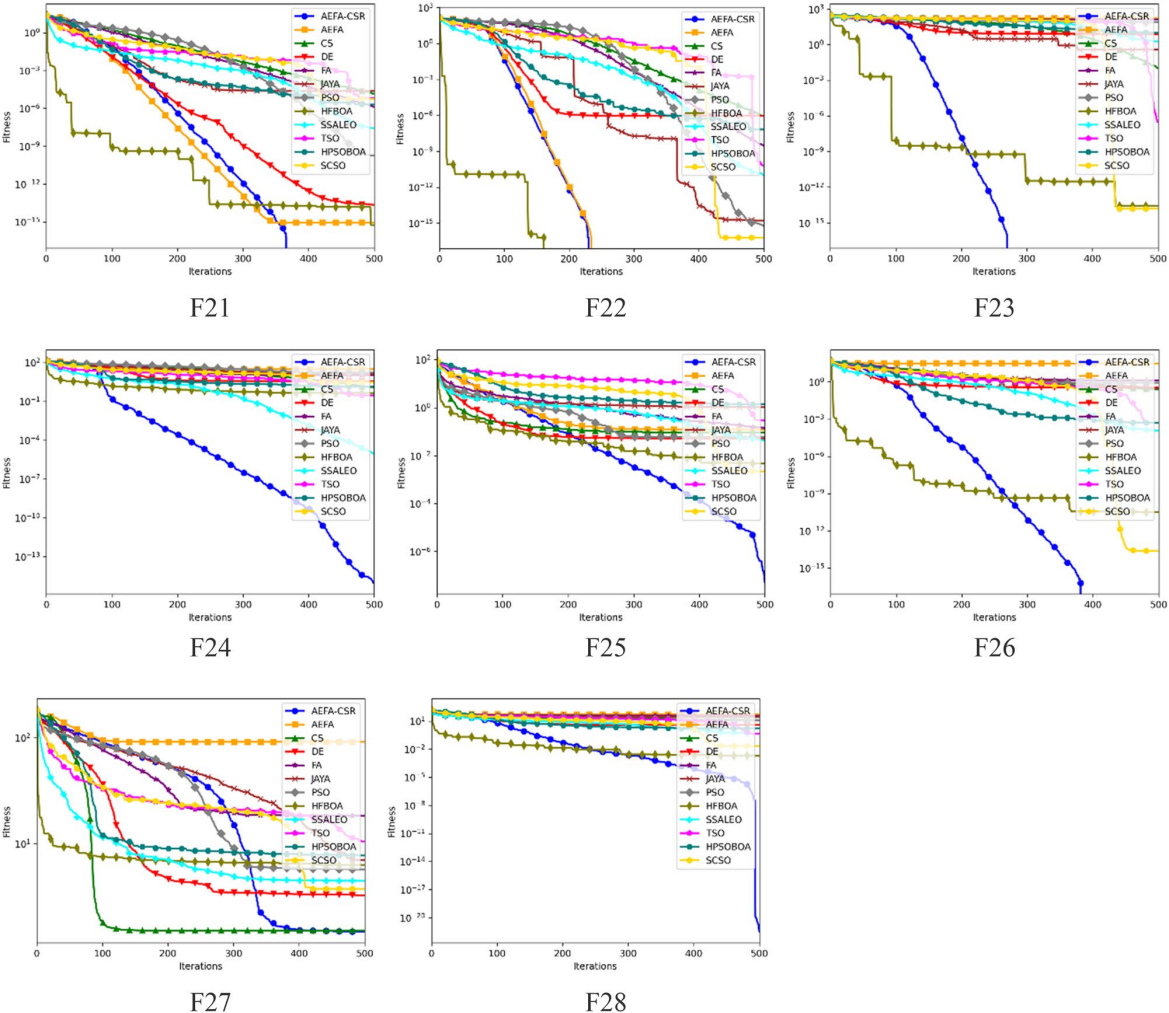
Convergence trajectory values for F21- F28.

The average (*Avg*) and standard deviation (*Std*) for each test function across 30 runs are shown in Table 14 and Fig. 6 displays their averaged convergence curves. In comparison to CS, PSO, DE, and JAYA, AEFA-CSR converges faster for F21 and F23 which represent single-antenna design problem. Also, obtaining the global optimal value for this functions suggests that AEFA-CSR is effective in addressing the single-antenna design problem. When compared to the other algorithms, AEFA-CSR has high efficiency for F22, F23 and F24 which depict multi-antenna properties and it is also able to escape the sub-optimality in F5. Because, multi-antenna problem typically exhibits the features of F22, F23, F24 and F25 concurrently. We may assume that AEFA-CSR is well equipped to solve them. The performance of AEFA-CSR for solving F26, F27 and F28 with the isolation characteristic of multi-antenna is comparable to that for F5. Refraction learning's ability to simulate complicated landscapes with several local extremums and steep, long, narrow valleys. CS improving variety in the population are the primary factors in AEFA-CSR's success.

**Table 14:** Test function optimization results for F21-F28.

		AEFA-CSR	AEFA	CS	DE	FA	PSO	JAYA	HFBOA	SSALEO	TSO	HPSOBOA	SCSCO
F21	Avg	**0**	9.00E−16	1.40E−5	2.34E−14	1.30E−6	1.74E−10	2.54E−5	5.78E−16	2.73E−8	6.42E−6	1.75E−8	5.97E−6
	Std	**0**	**0**	1.32E−6	**0**	2.15E−7	1.70E−10	1.75E−7	**0**	6.69E−10	**0**	9.62E−10	**0**
F22	Avg	**0**	**0**	9.26E−7	9.30E−7	3.00E−9	5.78E−16	1.67E−15	**0**	1.08E−11	6.40E−11	6.41E−14	6.42E−17
	Std	**0**	**0**	9.46E−8	**0**	2.05E−9	**0**	**0**	**0**	1.22E−13	7.61E−14	2.89E−15	**0**
F23	Avg	**0**	1.70E+2	1.03E−2	7.79	1.29E+2	8.48E+1	3.76E−1	2.64E−14	1.93	3.07E−7	6.24E−3	1.54E−14
	Std	**0**	5.93	6.67E−3	**0**	1.52	1.07	**0**	1.54E−14	1.68	1.35E−14	2.88E−5	**0**
F24	Avg	**9.64E−16**	3.03E+1	1.24	3.54	9.61	1.63E+1	1.21E+1	4.25E−1	7.80E−6	2.81E−1	1.63E−1	2.90
	Std	**0**	5.19	2.35E−4	5.35E−2	9.02E−5	6.97E−1	3.11E−3	1.18E−1	3.64E−6	1.48E−4	1.21E−1	5.58E−2
F25	Avg	**4.96E−8**	1.23E−1	9.01E−2	5.30E−2	1.42E−1	6.02E−2	1.05	4.77E−3	4.34E−2	2.88E−1	4.85E−1	2.25E−3
	Std	1.49E−8	6.36E−2	3.33E−2	2.95E−2	5.54E−2	**0**	3.16E−1	2.24E−4	1.00E−2	6.14E−4	4.03E−2	7.94E−4
F26	Avg	**0**	3.08E+1	2.47E−1	3.64E−1	1.36	8.74E−1	2.85E−1	3.18E−11	1.22E−4	5.00E−4	2.76E−6	2.52E−14
	Std	**0**	5.98	1.40E−1	1.22E−1	1.04	5.47E−6	1.48E−1	**0**	1.11E−5	7.51E−3	2.05E−7	**0**
F27	Avg	**1.47**	9.12E+1	1.51	3.26	1.84E+1	5.72	7.01	6.31	4.47	1.05E+1	6.84	3.75
	Std	**2.40E−1**	3.29E+1	2.06E−1	6.03E−1	2.74E−2	2.55E−2	2.87E−1	2.80E−2	5.93E−1	9.43E−1	5.26E−1	1.45
F28	Avg	**3.12E−22**	5.41E+1	1.27E+1	3.95	3.77E+1	1.24E+1	2.62E+1	2.08E−3	4.44E−1	4.65E−1	6.27E−1	2.22E−2
	Std	**3.03E−25**	3.13	4.22E−1	3.82E−2	2.86	3.25E−1	7.21	1.30E−4	1.46E−1	1.18E−2	4.50E−2	6.49E−6

Significant values are in bold.

#### Welded beam design problem

As a further validation of the performance of AEFA-CSR in real world optimization problem a well-known problem is chosen which is the welded beam design problem who was formulated by Rao^74^ and used in CEC 2020 test function suite^75^. The welded beam design problem has several design parameters as outlined in^55^. There are four design variables that need to be determined ${x}_{1},{x}_{2},{x}_{3}$ and ${x}_{4}.$ Under specific restrictions, the objective of WBD optimization is to reduce the overall cost. The restrictions are the buckling critical load $PC$, the bending stress $\sigma$, the shear stress $\tau$, beam deflection $\delta$ and the tail of the beam. The following are the objective function and constraints. The objective function which needs to be minimized is given below in Eq. (22).

22 $$f(\mathbf{x})=1.10471{x}_{1}^{2}{x}_{2}+0.04811{x}_{3}{x}_{4}\left(14+{x}_{2}\right)$$

The objective function is subject to the constraint equations given below (23) to (29).

23 $${g}_{1}\left(\mathbf{x}\right)=\sqrt{{\left({\tau }^{{^{\prime}}}\right)}^{2}+2{\tau }^{{^{\prime}}}{\tau }^{{^{\prime}}{^{\prime}}}\frac{{x}_{2}}{2R}+{\left({\tau }^{{^{\prime}}{^{\prime}}}\right)}^{2}}-{\tau }_{\mathrm{max}}\le 0$$

24 $${g}_{2}(\mathbf{x})=\frac{6PL}{{x}_{3}^{2}{x}_{4}}-{\sigma }_{\mathrm{max}}\le 0$$

25 $${g}_{3}\left(\mathbf{x}\right)={x}_{1}-{x}_{4}\le 0$$

26 $${g}_{4}\left(\mathbf{x}\right)=0.10471{x}_{1}^{2}+0.04811{x}_{3}{x}_{4}\left(14+{x}_{2}\right)-5\le 0$$

27 $${g}_{5}\left(\mathbf{x}\right)=0.125-{x}_{1}\le 0$$

28 $${g}_{6}\left(\mathbf{x}\right)=\frac{4P{L}^{3}}{E{x}_{3}^{3}{x}_{4}}-{\delta }_{\mathrm{max}}\le 0$$

29 $${g}_{7}(\mathbf{x})=P-\frac{4.013E{x}_{3}{x}_{4}^{3}}{6{L}^{2}}\left(1-\frac{{x}_{3}}{2L}\sqrt{\frac{E}{4G}} \right)\le 0$$

where

30 $${\tau }^{{^{\prime}}}=\frac{P}{2{x}_{1}{x}_{2}}$$

31 $${\tau }^{{^{\prime}}{^{\prime}}}=MRJ$$

32 $$M=P\left(L+\frac{{x}_{2}}{2}\right)$$

33 $$R=\sqrt{\frac{{x}_{2}^{2}}{4}+{\left(\frac{{x}_{1}+{x}_{3}}{2}\right)}^{2}}$$

34 $$J=2\left\{\sqrt{2}{x}_{1}{x}_{2}\left[\frac{{x}_{2}^{2}}{12}+{\left(\frac{{x}_{1}+{x}_{3}}{2}\right)}^{2}\right]\right\}$$

with the constants; $P=6000\mathrm{lb}$, $L=14\mathrm{in},E=30\times {10}^{6}\mathrm{psi},G=12\times {10}^{6}\mathrm{psi}$, ${\tau }_{\mathrm{max}}=\mathrm{13,600psi},{\sigma }_{\mathrm{max}}=30,000\mathrm{psi}, {\delta }_{\mathrm{max}}=0.25\mathrm{in}$.

The boundaries of the variables are given as;

$$0.1\le {x}_{1},{x}_{4}\le 2.0\text{ and }0.1\le {x}_{2},{x}_{3}\le 10.0$$

The results are compared with all the algorithms used for the experiments; AEFA, CS, DE, FA, PSO, JAYA, HFBOA, SSALEO, TSO, HPSOBOA and SCSCO. The population size is 30 and the algorithms are individually performed 20 times with a maximum of 500 iterations.

The best value of each optimization result for the twelve methods used to solve the welded beam design problem is shown in Table 15. Table 15 indicates that AEFA-CSR performs better than other methods on the optimization of welded beam design problem. It obtained the optimum value which is the least cost to be 1.695258.

**Table 15:** Welded beam design problem.

Algorithm	Optimal value	${x}_{1}$	${x}_{2}$	${x}_{3}$	${x}_{4}$
AEFA-CSR	1.695258	0.205734	3.253011	9.036754	0.205729
AEFA	1.796755	0.166780	4.190101	8.814214	0.216243
CS	1.861632	0.204408	3.800671	8.819535	0.223250
DE	1.696247	0.206011	3.249761	9.030545	0.206007
FA	1.716293	0.209133	3.225701	8.925939	0.210951
PSO	1.698182	0.206546	3.243466	9.018869	0.206541
JAYA	1.916665	0.211240	4.510793	8.883281	0.214170
HFBOA	1.699535	0.205755	3.283737	9.036164	0.205751
SSALEO	1.800743	0.150148	4.649707	8.946055	0.209916
TSO	1.698150	0.206111	3.245325	9.040353	0.206094
HPSOBOA	1.701435	0.207240	3.233837	9.009225	0.207237
SCSO	1.696550	0.206094	3.248803	9.028725	0.206090

#### Tension/compression spring optimization design problem

The tension/compression spring design problem’s optimization objective is to lower the spring weight^60^. It is a continuous constrained problem and the variables are wire diameter *d*, average coil diameter* D*, and effective coil number *P*. Constraints include subject to minimal deviation (*g*_1_), shear stress (*g*_2_), shock frequency (*g*_3_), and outside diameter limit (*g*_4_). The objective function and constrained equations are given below.

$$\mathbf{x}=\left[\begin{array}{lll}{x}_{1}& {x}_{2}& {x}_{3}\end{array}\right]=\left[\begin{array}{lll}d& D& P\end{array}\right]$$

35 $$f(\mathbf{x})={x}_{1}^{2}{x}_{2}\left(2+{x}_{3}\right)$$

36 $${g}_{1}(\mathbf{x})=1-\frac{{x}_{2}^{3}{x}_{3}}{71785{x}_{1}^{4}}\le 0$$

37 $${g}_{2}(\mathbf{x})=\frac{4{x}_{2}^{2}-{x}_{1}{x}_{2}}{\mathrm{12,566}\left({x}_{2}{x}_{1}^{3}-{x}_{1}^{4}\right)}+\frac{1}{5108{x}_{1}^{2}}\le 0$$

38 $${g}_{3}(\mathbf{x})=1-\frac{140.45{x}_{1}}{{x}_{2}^{2}{x}_{3}}\le 0$$

39 $${g}_{4}(\mathbf{x})=\frac{{x}_{1}+{x}_{2}}{1.5}-1\le 0$$

For decision variables, boundaries are given as,

$$0.05\le {x}_{1}\le 2.0, 0.25\le {x}_{2}\le 1.3 \text{and }2.0\le {x}_{3}\le 15.0$$

The results are compared with all the algorithms used for the experiments; AEFA, CS, DE, FA, PSO, JAYA, HFBOA, SSALEO, TSO, HPSOBOA and SCSCO. The population size is 30 and the algorithms are individually performed 20 times with a maximum of 500 iterations.

The findings in Table 16 show that each algorithm's weight is relatively low, which puts the algorithms' engineering problem-solving precision to the test. The AEFA-CSR produced the lowest weight as 0.012663 when the algorithms are taken into consideration.

**Table 16:** Tension/compression spring optimization design.

Algorithm	Optimal value	*d*	*D*	*p*
AEFA-CSR	0.012663	0.051685	0.356681	11.288568
AEFA	0.012684	0.052751	0.382863	9.903828
CS	0.013155	0.052257	0.367372	11.112651
DE	0.012925	0.050000	0.314312	14.448448
FA	0.013146	0.056171	0.424321	9.514575
PSO	0.012991	0.056046	0.470943	6.780858
JAYA	0.012738	0.050000	0.317535	14.037515
HFBOA	0.012827	0.054742	0.434764	7.843743
SSALEO	0.012734	0.053681	0.406623	8.865884
TSO	0.012750	0.050000	0.316953	14.090266
HPSOBOA	0.012717	0.050000	0.317478	14.019974
SCSO	0.013178	0.057170	0.503553	6.005638

In order to measure the effect of hybridization applied to AEFA, such tests are carried out; overall effectiveness in changing dimension and population, convergence analysis, Wilcoxon rank-sum and Friedman statistical tests, sensitivity, exploration and exploitation analyses and computational complexity. Additionally, it’s performance is validated through a set of real engineering design problems. In all analyses, it is depicted that CS significantly increase the population diversity while RL updates the lead agent. Therefore, it gets closer to the global optimum at each time which is a result of successfully built balance between exploration and exploitation.

## Conclusion and future work

This article proposes a solid optimizer AEFA-CSR that allows to solve engineering optimization problems with satisfactory performance. The comprehensive experimental analyses are conducted by including the commonly used, recently developed and hybrid algorithms on a benchmark test suite of 20 problems and three engineering design problems. It is well observed that the proposed algorithm AEFA-CSR is superior than the compared algorithms in terms of overall effectiveness. The algorithm’s performance for increasing population size is measured with the higher overall effectiveness in between 61.53 and 76.93%. When the dimension grows, the overall effectiveness is measured in between 76.93 and 90.0%. For the Wilcoxon Rank Sum statistical test for different control parameters combinations, AEFA-CSR attained the best performance than the other algorithms. However, in terms of computational time, since the algorithm is a combination of three separate methods, the results are not desirable as they are expected. Although the running time of AEFA-CSR is slightly more than the others, the running time is still acceptable and the algorithm produces more accurate and the efficient results than the others for all the functions analyzed. As a future work, the computational time results can be analyzed for further improvements. By considering the important contributions of AEFA-CSR, a balance might be built between the high accuracy and the computational time. Apart from this, it can be said with a confidence that the AEFA-CSR is a quite promising optimization algorithm and quite applicable in solving real-world engineering problems.

## Data Availability

The data obtained through the experiments are available upon a reasonable request from the first author O.R.A.
